# Full Genomic Sequences of H5N1 Highly Pathogenic Avian Influenza Virus in Human Autopsy Specimens Reveal Genetic Variability and Adaptive Changes for Growth in MDCK Cell Cultures

**DOI:** 10.1155/2021/3890681

**Published:** 2021-07-22

**Authors:** Kantima Sangsiriwut, Pirom Noisumdaeng, Mongkol Uiprasertkul, Jarunee Prasertsopon, Sunchai Payungporn, Prasert Auewarakul, Kumnuan Ungchusak, Pilaipan Puthavathana

**Affiliations:** ^1^Department of Preventive and Social Medicine, Faculty of Medicine Siriraj Hospital, Mahidol University, Bangkok 10700, Thailand; ^2^Faculty of Public Health, Thammasat University, Khlong Luang, Pathum Thani 12121, Thailand; ^3^Thammasat University Research Unit in Modern Microbiology and Public Health Genomics, Thammasat University, Thailand; ^4^Department of Pathology, Faculty of Medicine Siriraj Hospital, Mahidol University, Bangkok 10700, Thailand; ^5^Center for Research and Innovation, Faculty of Medical Technology, Mahidol University, Nakhon Pathom 73170, Thailand; ^6^Department of Biochemistry, Faculty of Medicine, Chulalongkorn University, Bangkok 10330, Thailand; ^7^Department of Microbiology, Faculty of Medicine Siriraj Hospital, Mahidol University, Bangkok 10700, Thailand; ^8^Bureau of Epidemiology, Ministry of Public Health, Nonthaburi 11000, Thailand

## Abstract

The entire H5N1 highly pathogenic avian influenza viral genomes were identified in the frozen autopsy specimens: the trachea, lung, colon, and intestinal feces from a patient who died of the disease in 2006. Phylogenetic analysis of the viral genomes showed that these viruses belonged to clade 1 and were the reassortants generated from the reassortment of the viruses within the same clade. The sequencing data from the autopsy specimens revealed at least 8 quasispecies of the H5N1 viruses across all 4 specimen types. These sequences were compared to those derived from the virus isolates grown in Madin Darby canine kidney (MDCK) cells. The virus isolates from the trachea, lung, and fecal specimens showed 27 nucleotide substitutions, leading to the changes of 18 amino acid residues. However, there was no change in the amino acid residues that determined the viral virulence. The changes were more commonly observed in the lung, particularly in the *HA* and *NA* genes. Our study suggested that the adaptation changes for the viral fitness to survive in a new host species (MDCK cells) might involve many genes, for example, the amino acid substitution 177G or 177W adjacent to the receptor-binding residues in the HA1 globular head and the substitution M315I in PB2. However, a mutation changes near the receptor binding domain may play an important role in determining the cell tropism and is needed to be further explored.

## 1. Introduction

Presently, the influenza A virus consists of 18 hemagglutinin (HA) and 11 neuraminidase (NA) subtypes. Viruses of various HA and NA combinations, i.e., between H1-H16 and N1-N9, are found in avian species [1, 2], while H17N10 and H18N11 are batlike viruses [2–4]. The subtypes that infect humans comprise H1N1, H2N2 (already extinct), and H3N2 viruses. The receptor preference for avian influenza (AI) viruses and human influenza viruses is different. Avian viruses prefer epithelial cell receptors containing *α*2,3-galactose linked-sialic acid (*α*2,3 gal-SA). In contrast, human viruses prefer epithelial cells containing *α* 2,6 galactose linked-sialic acid (*α* 2,6 gal-SA) [5]. Most AI viruses are of low pathogenicity and cause asymptomatic or mild infection in avian species, but some strains of H5 and H7 are highly pathogenic. Occasionally, some avian viruses cross the species barrier to infect human. The fatality rate of seasonal influenza viruses in humans is generally minimal, while that of avian viruses in humans is as high as 33% for the H5N1 highly pathogenic avian influenza (HPAI) viruses that first emerged in Hong Kong in 1997 [6, 7], 53% worldwide for H5N1 HPAI viruses that have reemerged since 2003 [8], and 39% for H7N9 virus [9]. Thailand first reported an H5N1 AI outbreak in poultry and humans in January 2004. The last human case was reported in 2006, while it was 2008 for the last poultry outbreak. There were a total of 25 human cases with 17 deaths or a fatality rate of 68% [8].

The pathology of human influenza and H5N1 HPAI is markedly different. Human influenza virus infections are confined mainly to the respiratory tract, while H5N1 HPAI virus infections spread beyond the respiratory tract to distal end organs [10]. Previous investigators demonstrated the presence of both genomic RNA and antigenomic RNA in various organs, including the lung, intestine, spleen, trachea, brain, heart, liver, kidneys, and lymph nodes [11–15]. The H5N1 virus has never been isolated from human specimens outside of the respiratory tract, except those in our study. Moreover, we also announced the discovery of full-genome sequences of H5N1 HPAI virus in archival organ tissues of a dead case by next-generation sequencing (NGS) technique [16], but the sequence analysis has not been performed yet. In this study, the amino acid sequences of various viral genes from the autopsy specimens were aligned with those from the virus isolates, and the substitutions that may be associated with the viral adaptation for replication in a new host species were determined.

## 2. Materials and Methods

### 2.1. Ethical Issues

This study was approved by the Institutional Review Board, Faculty of Medicine Siriraj Hospital, Mahidol University, Thailand.

### 2.2. An H5N1 Patient and Clinical Specimens

In August 2006, a 59-year-old man who lived in Nong Bua Lam Phu Province, Northeast of Thailand, was hospitalized with severe pneumonia which later progressed to acute respiratory distress syndrome and multiorgan failure. Several attempts to diagnose H5N1 avian influenza by genome detection failed. Nevertheless, he was treated with a standard regimen of oseltamivir on day 16 after the onset of symptoms when a history of contact to sick and dead chickens was obtained. He died of the disease at day 28 after the onset. Autopsy tissues and intestinal fecal samples were collected for disease diagnosis by viral genome detection, virus isolation in MDCK cells, and indirect immunofluorescence assay (IFA). The remaining fresh tissues were stored frozen at -80°C.

### 2.3. Laboratory Investigation

Each tissue (the trachea, lung, colon, spleen, and liver) was washed and processed individually with separate sets of instruments. Conventional reverse transcription-polymerase chain reaction was performed using the protocol described by Poddar [17] and the World Health Organization [18]. However, the results were inconclusive with all tissue samples investigated, including the intestinal fecal sample. An indirect immunofluorescence assay (IFA) was carried out by staining the impression smears of tissues with a monoclonal antibody to the influenza A nucleoprotein (Chemicon), and positive results were obtained with the trachea and lung epithelial cells.

Tissues samples (the trachea, lung, colon, spleen, and liver) were ground in viral growth media (VGM) containing Earle's Minimal Essential Medium (EMEM, ThermoFisher) without fetal bovine serum supplementation, and the intestinal fecal samples was suspended in VGM. The specimen suspensions were centrifuged, and the supernatants were inoculated onto MDCK cell monolayers maintained in VGM. Three serial blind passages were carried out, and virus isolation was successful with samples from the trachea, lung, and fecal samples. These H5N1 virus isolates were named A/Thailand/NBL1/2006-L, A/Thailand/NBL1/2006-T, and A/Thailand/NBL1/2006-F, for the lung, trachea, and fecal specimen origin, respectively. The virus isolates at the 4^th^ passage were subjected to full genome sequencing by the Sanger method, and the sequencing data was deposited in the GenBank database (Table 1).

### 2.4. Next-Generation Sequencing of Direct Autopsy Specimens

NGS was conducted on tissue samples after long-term storage for almost 10 years at -80°C. Total RNA was extracted from the trachea, lung, colon, and fecal samples using TRIzol Reagent (Invitrogen) and were cleaned using the RNeasy Mini Kit (Qiagen). The extracted RNA was reverse transcribed into cDNA using the SuperScript III First-Strand Synthesis System (Invitrogen). The cDNA was then used as a template to amplify all 8 segments of the influenza viral genome using Platinum Taq HiFi (Invitrogen). The entire genomic segment of PB2, PB1, PA, HA, NP, and NA (>1 kb) were amplified as overlapping fragments, namely, PB2a, PB2b, PB1a, PB1b, PAa, PAb, HAa, HAb, NPa, NPb, NAa, and NAb, respectively. The M and NS genomic segments were amplified as a single segment. The nucleotide sequences of these sequencing primers can be obtained on request. A 50 *μ*l volume PCR reaction consisted of 5 *μ*l of 10x PCR buffer, 2 *μ*l of 50 mM MgSO_4_, 1.5 *μ*l of 10 mM dNTPs, 2 *μ*l of each 10 *μ*M forward and reverse primers, 0.2 *μ*l of Platinum Taq HiFi, 5 *μ*l of cDNA, and distilled water. The PCR was carried out in a GeneAmp PCR system 2400 thermal cycler (Applied Biosystems). The reaction was composed of 1 cycle of holding at 94°C for 5 minutes, followed by 35 cycles of amplification (94°C for 15 seconds, 55°C for 30 seconds, and 68°C for 2 minutes), and a final extension step at 68°C for 10 minutes. The amplified PCR products were then purified using the QIAquick Gel Extraction kit (Qiagen). Full-genome sequencing was performed in three steps: library preparation, emulsion PCR (emPCR), and high-throughput sequencing using the GS Junior platform (Roche Diagnostics). Briefly, 500 ng of each amplified DNA fragments including PB2a, PB2b, PB1a, PB1b, PAa, PAb, HAa, HAb, NPa, NPb, NAa, Nab, M, and NS were pooled together and ligated to the adapters followed by emulsion PCR and sequencing. Four DNA libraries with different barcodes obtained from the trachea, lung, colon, and fecal samples were generated by using the GS DNA Rapid Library Preparation kit (Roche) according to the manufacturer's protocol.

### 2.5. NGS Data Analysis

Raw sequencing data were classified into each sample based on the barcode sequences within the DNA libraries. Secondary data analysis was performed using the CLC Genomics Workbench version 8.0.1 (http://www.clcbio.com/). Low-quality data (*Q* score < 30) and the adaptor sequences were trimmed and removed from the sequencing reads. The passed filter (PF) reads (*Q* score > 30) were used for mapping and alignment with the influenza reference genome of A/Thailand/1(KAN-1)/2004 (H5N1) (GenBank accession no. AY555144-AY555151). Each specimen's total reads ranged between 100,000 and 200,000, and the number of mapped reads ranged between 98% and 99%. The average read length for all samples ranged between 241 and 309 base pairs. The viral sequences obtained from each tissue were deposited in GenBank under the accession numbers MG668904 to MG668911 for the colon, MG668920 to MG668927 for the trachea, MG668928 to MG668935 for the lung, and MG668912 to MG668919 for the fecal specimen as shown in Table 1. The raw reads were deposited in the Sequence Read Archive under the BioProject accession number PRJNA494792 [16]. Nucleotide variations were examined and calculated for percentages of minor mutations in each gene. The genome signature corresponding to the relative frequency of amino acid residues for each gene was graphical generated using WebLogo (https://weblogo.berkeley.edu/logo.cgi) [19]. The prediction of mutational positions on the HA protein structure was investigated using a model of PDB code: 3FKU [20] and analyzed using UCSF Chimera program version 1.13.1 [21].

### 2.6. Phylogenetic Tree Construction

The reference strains with known genetically clades were retrieved from the WHO (https://www.who.int/influenza/gisrs_laboratory/h5n1_nomenclature/en/) [22] and NCBI GenBank databases (https://www.ncbi.nlm.nih.gov). The concatenated entire coding sequence (CDS) from 8 segments (PB2, PB1, PA, HA, NP, NA, M, and NS) and individual segments of H5N1 viruses isolated in Thailand were phylogenetically analyzed. The sequences were aligned using ClustalW multiple sequence alignment in BioEdit program version 7.0.4.1, and a phylogenetic tree was constructed using MEGA 7.0 software (http://megasoftware.net/) [23]. The evolutionary distances were estimated by using the neighbor-joining method and maximum composite likelihood algorithm. The reliability of the neighbor-joining tree was estimated by bootstrapping analysis using 1,000 replicate datasets. The supporting bootstrap value of greater than 80% was shown at the nodes of each cluster.

### 2.7. Selection Pressure Analysis

Human H5N1 and H5N6 viruses isolated between 1997 and 2018 were analyzed across clades for the ratio of nonsynonymous (dN) to synonymous (dS) substitution (dN/dS) on a codon-by-codon basis using the estimate selection for each codon (HyPhy) application under the model of Hasegawa-Kishino-Yano method in MEGA 7.0 software [23].

## 3. Results

### 3.1. Heterogeneity of H5N1 Influenza Viral Genomes in Various Tissue Organs

This study explored the H5N1 influenza viral genomes in four kinds of autopsy specimens: the trachea, lung, colon, and intestinal fecal specimen by NGS. The results showed that the H5N1 viral genome was detected in all of the specimens investigated. Deep sequencing analyses of the entire genome in every tissue demonstrated the virus quasispecies. An alignment of the viral nucleotide sequences from these 4 specimens showed 8 quasispecies in 6 gene segments, i.e., PB2, PB1, PA, HA, NP, and NA, but not in M and NS. Four positions of mixed nucleotide residues were present in the PB2 segment. Mixing A and G was found at 7 positions, while mixing T and G was found at 1 position (Table 2 and Fig. S1).

### 3.2. Virus Adaptation for Growth in MDCK Cells

**Figure 1:**
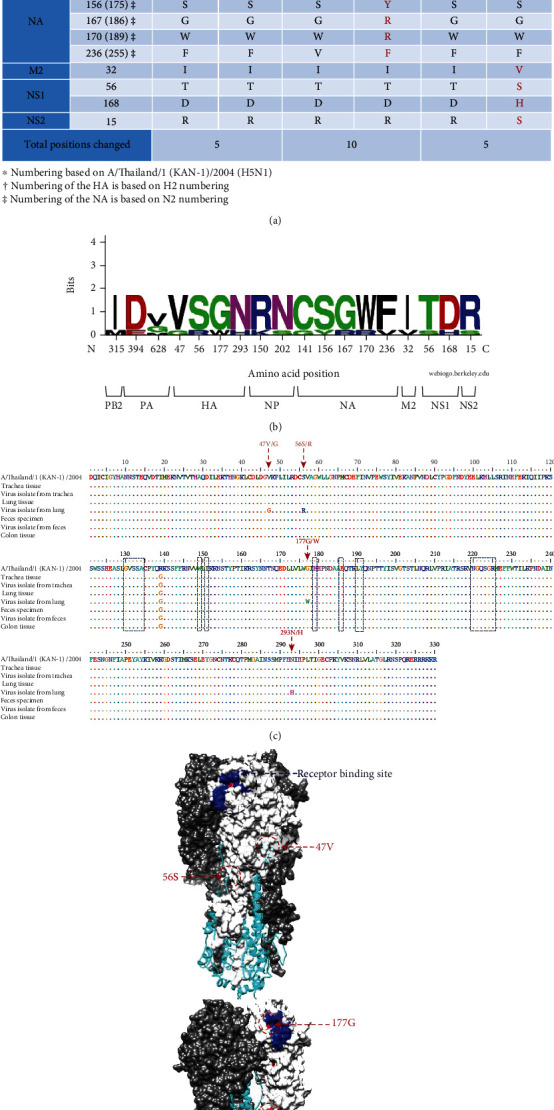
Genetic variability and genome signature in the H5N1 viral. (a) Amino acid changes as compared between amino acid sequencing from the autopsy specimens and the virus isolates. (b) The genomic signatures of amino acid residues obtained from an alignment. The graphical presentation was performed using WebLogo. The height of symbol represents the relative frequency and proportion of the amino acid residue corresponding at the particular position. (c) An alignment of the HA1 amino acid sequences. (d) The 3D structure of trimeric HA molecule (PDB code: 3FKU) showing the point mutation position observed in the study.

Three influenza virus isolates derived from the trachea, lung, and fecal samples at the 4^th^ passage in MDCK cells were sequenced by the Sanger method. The sequencing data have been deposited in the GenBank database since 2009 with the accession numbers shown in Table 1. The sequences of all 8 viral genes of these isolates were aligned with those derived from the corresponding specimens determined by NGS. The result on amino acid sequence analysis showed 27 nucleotide changes, resulting in 18 amino acid substitutions across 7 genomic segments: PB2, PA, HA, NP, NA, M, and NS, as shown in Figures 1(a) and 1(b) and Fig S2.

The genome signature of the viruses found in the autopsy specimens demonstrated the frequency or proportion of amino acid variations corresponding to the point mutations found in the virus isolates (Figure 1(b)). Most variations were more frequent with the lung isolate, particularly in the HA and NA gene segments. Four amino acid changes were observed in the HA1 domain, which contained the receptor binding site (RBS) for viral attachment to the host cell surface, determining the host cell tropism or specificity. The alignment of HA1 amino acid sequences derived from the autopsy specimens, clinical isolates, and A/Thailand/1(KAN-1)/2004 (H5N1) clade 1 virus showed that the point mutations 177G or 177W were adjacent to the receptor binding residues 179H, 186E, 190L, and 191Y (Figure 1(c)). The amino acid position 177 was in the globular head closed to the RBS, as demonstrated by the 3D-crystalized structure (Figure 1(d)). Sequence variation or genetic drift on the HA1 domain might influence the receptor binding, adaptive change for host cell tropism, and affected the viral antigenic epitopes. We also found the nucleotide 945G in PB2 of the trachea tissue, while 945A was found in the lung, colon, and fecal specimens (Fig. S1). This led to the nonsynonymous mutation of M315I in the virus isolate from the trachea. This M315I amino acid change may be necessary for adaptation to grow in MDCK cells since the 315I was found in virus isolates from all kinds of clinical specimens.

### 3.3. H5N1 Virus Clade Identification

**Figure 2:**
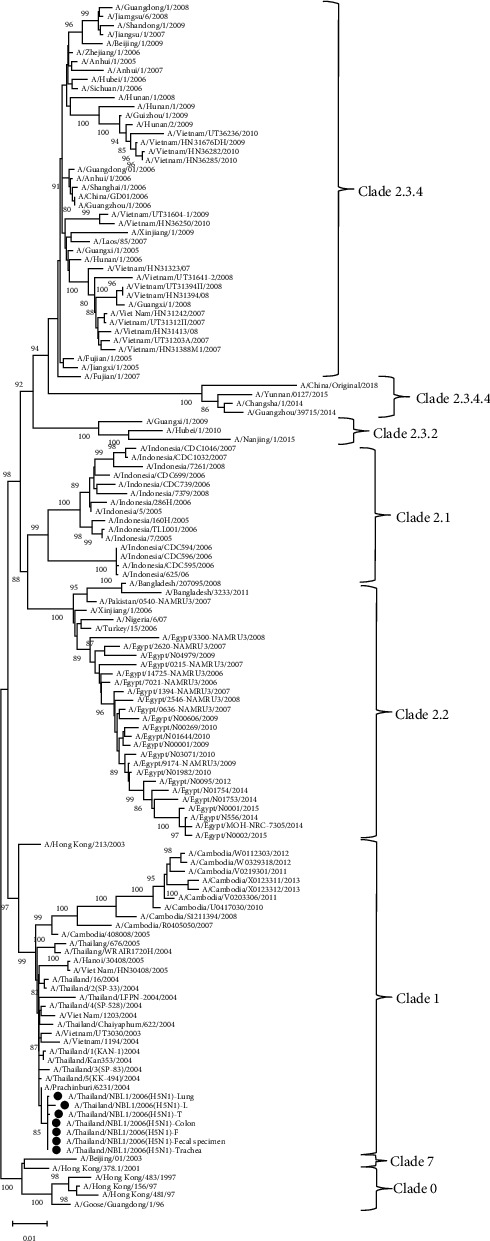
Phylogenetic analysis of H5 clades based on HA nucleotide sequences. The HA nucleotide sequences of our H5N1 viruses (● black circle) were analyzed for clade identification against various WHO reference H5 avian influenza strains that caused human infections: H5N1 virus clades 0, 1, 2.1, 2.2, 2.3.2, and 2.3.4 and the H5N6 virus clade 2.3.4.4.

**Figure 3:**
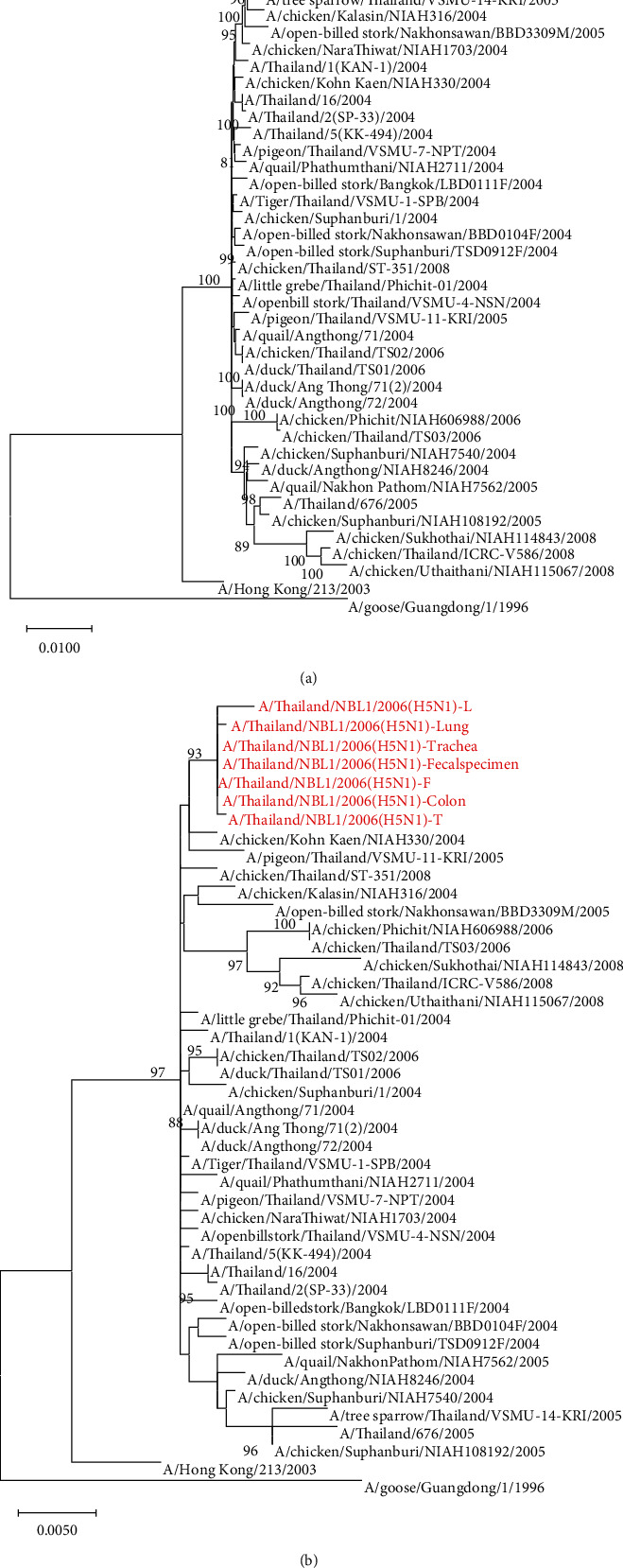
Phylogenetic analyses of the concatenated entire coding sequence of 8 segments (PB2, PB1, PA, HA, NP, NA, M, and NS) (a) and HA segment (b).

The HA nucleotide sequences of our H5N1 viruses were analyzed for clade identification against various WHO reference H5 avian influenza strains that caused human infections: H5N1 virus clades 0, 1, 2.1, 2.2, 2.3.2, and 2.3.4 and the H5N6 virus clade 2.3.4.4 [24, 25]. The phylogenetic analysis demonstrated that our viruses belonged to genetic clade 1 and were closely related to other H5N1 isolates in Thailand, Vietnam, and Cambodia (Figure 2). Furthermore, the concatenated complete coding sequences of 8 segmented genomes: PB2, PB1, PA, HA, NP, NA, M, and NS, and the HA segments were genetically analyzed against the H5N1 viruses circulating in Thailand in various host species from 2004 to 2008. The result showed that the concatenated PB2, PB1, PA, HA, NP, NA, M, and NS segments of A/Thailand/NBL1/2006 viruses mostly resembled A/Tree sparrow/Thailand/VSMU-14-KR/2005 clade 1 virus, while the HA genomic sequences mostly resembled A/Chicken/Kohn Kaen/NIAH330/2004 clade 1 virus (Figure 3). The finding led to the speculation that our viruses were reassortants. We then further phylogenetically analyzed each gene segment individually. The result confirmed that these H5N1 viruses were reassortants, which obtained 7 gene segments: PB2, PB, PA, NP, NA, M, and NS from A/Tree sparrow/Thailand/VSMU-14-KR/2005-like virus and the HA gene segment from A/Chicken/Kohn Kaen/NIAH330/2004-like virus (Fig. S3).

### 3.4. Genetic Variability across H5 Virus Clades

The HA amino acid sequences across clades of human H5N1 and H5N6 viruses circulating from 1997 to 2018 were compared. The result showed the genetic variability at positions 47, 56, 177, and 293 in our lung virus isolate. In contrast, the HA sequences from all 4 autopsy specimens and the virus isolates from tracheal and fecal specimens were highly conserved. However, the estimating ratio of the nonsynonymous (dN) to synonymous (dS) substitutions (dN/dS) of <1 indicated the negative or purifying selective pressure of these positions across the genetic clades on a codon-by-codon basis (Table 3).

### 3.5. Analysis for H5 Virulence Determinants

Amino acid residues in the autopsy specimens and the virus isolates were analyzed following the H5N1 genetic change inventory [26], as demonstrated in Table 4. The result showed that the amino acid sequences in all autopsy specimens and the virus isolates contained PQRERRRKKRG at the HA cleavage site, indicating their high virulence. The residues 190E, 225G, 226Q, 227S, and 228G (H3 numbering), indicated that all viruses preferred the *α*2,3-galactose linked-sialic acid avian type receptor. The H274Y substitution, which indicated the oseltamivir and peramivir resistance, was not present in both the autopsy specimens and the virus isolates, even though the patient received a full course of oseltamivir treatment before his death. On the other hand, both the autopsy specimens and the virus isolates contained the S31N substitution in the M2 protein, indicating the amantadine and rimantadine resistance.

## 4. Discussion

H5 HA, in combination with various NA subtypes, were the most frequent HPAI viruses that cause human infections. The most common subtype, the H5N1 virus, has caused human infections in 16 countries, resulting in 862 cases with 455 deaths or a fatality rate of 52.8% after its reemergence in 2003 until 9 December 2020. The latest human H5N1 virus-infected case occurred in the Lao People's Democratic Republic (Lao PDR) and was reported to the WHO on 31 October 2020. The human H5N6 virus infection was less common with the latest case reported on 1 December 2020 from China [8]. Recently, the first human H5N8-infected case was reported in Russia in December 2020 [27].

Even though the information on the pathology of H5N1 fatal cases is quite limited, the concordant results suggested that the H5N1 HPAI virus could disseminate and replicate in organs beyond the respiratory tract [13, 28, 29]. Furthermore, the level of natural intrinsic heterogeneity, mutation frequencies, and single-nucleotide polymorphisms (SNPs) of influenza A viruses was previously reported by several investigators [30, 31]. With the NGS technique available lately, this study could demonstrate the complete H5N1 genomic sequences in all 4 kinds of autopsy specimens: the trachea, lung, colon, and intestinal feces. The nucleotide sequence alignment showed 8 viral quasispecies with 6 gene segments, i.e., PB2, PB1, PA, HA, NP, and NA, but not in M and NS. The NGS technique provided in-depth information on the natural amino acid residues present in the viral genomes in each autopsy specimen.

Our previous attempts to isolate the virus from various autopsy tissues of a few H5N1 patients did not succeed. Failure to isolate the virus from tissues that contain influenza viral genomes is not well understood. The infectious viruses might be destroyed by the tissue reactions resulting from a cytokine storm at the end stage of the disease. However, our attempt to isolate the virus was successful in the study patient who died of H5N1 HPAI virus infection in 2006. The H5N1 viruses were isolated from the trachea, lung, and intestinal fecal samples, but not the other organs. The nucleotide/amino acid sequences of these virus isolates were aligned against those of the autopsy specimens. A total of 27 nucleotide changes resulting in 18 amino acid substitutions were found. Mutational change(s) found in our virus isolates occurred during passaging in MDCK cells which had been going on until the 4^th^ passage before sequencing. Our study showed that the mutational change was more frequent with the lung isolate and, in particular, in the HA and NA genes. This finding goes along well with basic influenza virology that HA and NA genes are highly variable compared to the other genes. There were 4 amino acid changes in the HA protein of our lung virus isolates that were not present in the other viruses in this study and other virus clades. However, the determination for natural selection showed the dN/dS of <1, which suggested the negative selection pressure of these residues.

Our study found the point mutations 177G in viruses from the trachea and fecal specimens and 177W from the lung virus. According to the 3D structure, the amino acid position 177 did not locate on the external surface of HA1 globular head; nevertheless, it was adjacent to the receptor-binding residues 179H, 186E, 190L, and 191Y. We suggested that the amino acid change at position 177 may not directly responsible for binding to sialic acid host cell receptors. Nevertheless, we cannot exclude the possibility of the steric hindrance phenomenon arose from the mutational change at amino acid position 177 that might affect its neighboring amino acid residues in binding to the receptor biding site. Moreover, we showed that the M315I amino acid change may be essential for the trachea virus to adapt for growth in MDCK cells since the 315I was found in virus isolates from all types of clinical specimens. Our study suggested that the changes of amino acid residues in multiple genes might be required for viral adaption to grow in a new host species. All virus isolates of different origins could grow in MDCK cells and yielded comparable titers to the other H5N1 viruses in our laboratory.

Phylogenetic analyses relying on the concatenated PB2, PB1, PA, HA, NP, NA, M, and NS genes or each gene suggested that our H5N1 viruses were the reassortants generated from two clade 1 viruses: A/Tree sparrow/Thailand/VSMU-14-KR/2005-like virus and A/Chicken/Kohn Kaen/NIAH330/2004-like virus. Our group and the other Thai investigators reported the reassortants generated from two H5N1 clade 1 viruses in avian hosts [32, 33]. In this study, we reported the reassortant H5N1 virus infection in a Thai patient. Nevertheless, the reassortant H5N1 virus infection in humans had been previously reported in Vietnam [34, 35] and Cambodia [36].

Furthermore, the amino acid substitution S31N in the M2 protein of our viruses suggested that the virus was resistant to amantadine and rimantidine [37], and the 274H residue in the NA protein suggested that the viruses were susceptible to oseltamivir. This patient died because the oseltamivir was prescribed late, and additionally, he was superimposed to *Acinetobacter baumannii* infection. This study also analyzed for virulence determinants present in the H5N1 HPAI viral genomes based on the Center for Disease Prevention and Control guidelines [26]. Our viral genomes contained amino acid residues 89V, 309D, 339K, 477G, 495V, 676T, and 627K in PB2 and residues 30D and 215A in M1, all of which are related to enhance viral virulence, increased polymerase activity, or increased replication efficiency. In contrast, our viral genomes also contained the residues 598L in PB1 and 149S and 357I in PA that are related to decreased polymerase activity and replication efficiency. Nevertheless, there was no change in the amino acid residues that determine the H5N1 HPAI viral virulence, particularly, the presence of multiple basic amino acids at the HA cleavage site and the 20 amino acid deletion in NA. The adaptation change for survival in a new host species might involve many genes. Still, the HA and NA genes that determine cell tropism and virulence determinants are likely to be more important than the others.

## 5. Conclusion

H5N1 avian influenza virus disseminated and infected the organs beyond the respiratory tract. This study was the first to report the complete viral genomes in the archival specimens from an autopsy: the lung, trachea, colon, and intestinal fecal samples. The H5N1 virus was isolated from the lung, trachea, and fecal samples using MDCK cells. The phylogenetic analysis demonstrated that this virus was a reassortant in which the HA segment from one avian species reassorted with the 7 segments of another virus from different avian species. The study also found novel amino acid substitutions.

## Figures and Tables

**Table 1:** Demographical patient information and H5N1 viral genomic sequences.

Patient	Location	Virus origin^∗^^1^	Virus name	Segment	GenBank accession no.
A 59-year-old man	Nong Bua Lam Phu Province, northeast of Thailand	Autopsy tissue organs	A/Thailand/NBL1/2006 (H5N1)-Lung	PB2, PB1, PA, HA, NP, NA, M, NS	MG668928-MG668935
			A/Thailand/NBL1/2006 (H5N1)-Trachea	PB2, PB1, PA, HA, NP, NA, M, NS	MG668920-MG668927
			A/Thailand/NBL1/2006 (H5N1)-Fecal specimen	PB2, PB1, PA, HA, NP, NA, M, NS	MG668912-MG668919
			A/Thailand/NBL1/2006 (H5N1)-Colon	PB2, PB1, PA, HA, NP, NA, M, NS	MG668904-MG668911
		Virus isolates	A/Thailand/NBL1/2006 (H5N1)–L^∗^^2^	PB2, PB1, PA, HA, NP, NA, M, NS	GQ466176-GQ466183
			A/Thailand/NBL1/2006 (H5N1)–T^∗^^3^	PB2, PB1, PA, HA, NP, NA, M, NS	KJ907475-KJ907482
			A/Thailand/NBL1/2006 (H5N1)–F^∗^^4^	PB2, PB1, PA, HA, NP, NA, M, NS	KJ907467-KJ907474

Note: ^1^full-genome sequencing from direct specimens was obtained by using next-generation sequencing (NGS) with the GS Junior platform (Roche Diagnostics, Basel, Switzerland), meanwhile full-genome sequencing from virus isolates were obtained by Sanger DNA sequencing method. ^2^Virus isolate from the lung. ^3^Virus isolate from the trachea. ^4^Virus isolate from the fecal specimen.

**Table 2:** Nucleotide heterogeneities of HPAI H5N1 viral genomes in autopsy specimens as demonstrated by NGS.

Segment	Nucleotide position^∗^^1^	Type of autopsy specimens				Amino acid change
		Trachea	Lung	Feces	Colon	
PB2	945	G	A	A	A	M315I
PB1	549	A (63%) G (37%)	A (61%) G (39%)	A (51%) G (49%)	G (52%) A (48%)	—
PA	1882	A	G	G	G	M628V
HA	1230	G	A (62%) G (38%)	G	G	—
NP	449	A (53%) G (47%)	G	G	G	K150R
	605	G (59%) A (41%)	A	A	A	S202N
NA	399 (459)^∗^^2^	T	C	C	T	—
	706 (763)^∗^^2^	T	G (53%) T (47%)	T	T	V255F
	723 (780)^∗^^2^	A	A	G	A	—

Note: ^1^nucleotide numbering based on influenza A/Thailand/1(KAN-1)/2004 (H5N1). ^2^NA numbering based on N2 subtype.

**Table 3:** Amino acid comparison with various H5 clades and maximum likelihood analysis of natural selection.

H5 virus clade	HA amino acid position^∗^^1^			
	47 (56)^∗^^2^	56 (65)^∗^^2^	177 (181)^∗^^2^	293 (296)^∗^^2^
Clade 0 (*n* = 4)	V	S	G	N
Clade 1 (*n* = 27)	V	S	G	N
Clade 2.1 (*n* = 15)	V	S	G	N
Clade 2.2 (*n* = 29)	V	S	G	N
Clade 2.3.2 (*n* = 3)	V	S	G	N
Clade 2.3.4 (*n* = 42)	V	S	G	N
Clade 2.3.4.4 (*n* = 4)	V	S	G	N
A/Thailand/NBL1/2006 (H5N1)-Lung	V	S	G	N
A/Thailand/NBL1/2006 (H5N1)-Trachea	V	S	G	N
A/Thailand/NBL1/2006 (H5N1)-Fecal specimen	V	S	G	N
A/Thailand/NBL1/2006 (H5N1)-Colon	V	S	G	N
A/Thailand/NBL1/2006 (H5N1)–L^3^	G	R	W	H
A/Thailand/NBL1/2006 (H5N1)–T^4^	V	S	G	N
A/Thailand/NBL1/2006 (H5N1)–F^5^	V	S	G	N
dN-dS	-3.5	-2.0	-4.5	-5.7
dN/dS	0.125	0.186	0.100	0.073
*P* value	0.995	0.980	0.998	0.999

Note: ^1^numbering based on A/Thailand/1(KAN-1)/2004 (H5N1). ^2^Numbering of the HA is based on H3 numbering. ^3^Virus isolate from lung. ^4^Virus isolate from trachea. ^5^Virus isolate from fecal specimen.

**Table 4:** Analysis for virulence determinants of H5N1 HPAI virus in this study compared to the H5N1 genetic change inventory.

Protein	Amino acid mutation previously reported	Association and function	Amino acid observed in autopsy/culture
PB2	I63T	Decreased pathogenicity in mice	63I
	E627K	Increased replication efficiency in cell culture and enhanced virulence in mice	627K
		Enhanced polymerase activity and mammalian host adaptation	
		Mammalian host adaptation, increased virulence in mice	
		Enhanced polymerase activity	
		H5 virus transmissible among ferrets	
	M28I, A274T, K526R, I553V, L607V	Decreased polymerase activity	28M, 274A,526K, 553I, 607L
	L89V, G309D, T339K, R477G, I495V, A676T	Enhanced polymerase activity, increased virulence in mice	89V, 309D, 339K, 477G, 495V, 676T
PB1	K207R	Decreased polymerase activity in mammalian cells	207K
	Y436H	Decreased polymerase activity and virulence in mallards, ferrets, and mice	436Y
	T677M	Decreased virulence in mice	677T
	V3A, N328K, N375S	Decreased replication efficiency and virulence in ferrets	3V, 328N, 375N
	V473L, P598L	Decreased polymerase activity and replication efficiency	473V, 598L
PA	P149S, R266H, K357I, T515S	Decreased polymerase activity in mammalian cells	149S, 266R, 357I, 515T
NA	49-68 deletion	Enhanced virulence in mice	49-68 deletion
M1	N30D, T139A, T215A	Increased virulence in mice	30D, 139T, 215A
M2	L26F, V27A, G34E, A30V/T/S, S31N/G	Reduced susceptibility to amantadine and rimantadine	26I, 27V, 34G, 30A, 31 N
NS1	80-84 deletion, P42S, D87E, L98F, L101M	Increased virulence in mice	80-84 deletion,42S, 87D, 98F, 101M
	ESEV (in PDZ domain)	Increased virulence in mice	ESEV(in PDZ domain)

## Data Availability

All data generated or analyzed are included within the manuscript and supplementary materials.

## Supplementary Materials

Supplementary 1 Supplementary Figure S1: multiple alignments of the nucleotide sequences of H5N1 HPAI viral genomes in the autopsy specimens sing NGS. The genes containing variations/quasispecies are shown in open boxes.

Supplementary 2 Supplementary Figure S2: multiple alignments of the amino acid sequences of H5N1 HPAI virus derived from the autopsy specimens and the virus isolates. The amino acid changes are shown in open boxes.

Supplementary 3 Supplementary Figure S3: phylogenetic analyses of an individually remaining 7 gene segments including PB2, PB1, PA, NP, NA, M, and NS from clade 1 H5N1 viruses in Thailand.

## References

[1] Dugan V. G., Chen R., Spiro D. J. (2008). The evolutionary genetics and emergence of avian influenza viruses in wild birds. *PLoS Pathogens*.

[2] Yoon S. W., Webby R. J., Webster R. G. (2014). Evolution and ecology of influenza A viruses. *Influenza Pathogenesis and Control - Volume I*.

[3] Tong S., Li Y., Rivailler P. (2012). A distinct lineage of influenza A virus from bats. *Proceedings of the National Academy of Sciences*.

[4] Tong S., Zhu X., Li Y. (2013). New world bats harbor diverse influenza A viruses. *PLoS Pathogens*.

[5] Suzuki Y. (2005). Sialobiology of influenza: molecular mechanism of host range variation of influenza viruses. *Biological & Pharmaceutical Bulletin*.

[6] Claas E. C., Osterhaus A. D., van Beek R. (1998). Human influenza A H5N1 virus related to a highly pathogenic avian influenza virus. *Lancet*.

[7] Hatta M., Kawaoka Y. (2002). The continued pandemic threat posed by avian influenza viruses in Hong Kong. *Trends in Microbiology*.

[8] World Health Organization Cumulative number of confirmed human cases for avian influenza A (H5N1) reported to WHO, 2003-2021. https://www.who.int/publications/m/item/cumulative-number-of-confirmed-human-cases-for-avian-influenza-a(h5n1)-reported-to-who.

[9] Food and Agriculture Organization of the United Nations 2021. H7N9 situation update. http://www.fao.org/ag/againfo/programmes/en/empres/H7N9/situation_update.html.

[10] NG W., TO K., LAM W., NG T., LEE K. (2006). The comparative pathology of severe acute respiratory syndrome and avian influenza A subtype H5N1--*a* review. *Human Pathology*.

[11] Uiprasertkul M., Puthavathana P., Sangsiriwut K. (2005). Influenza A H5N1 replication sites in humans. *Emerging Infectious Diseases*.

[12] Uiprasertkul M., Kitphati R., Puthavathana P. (2007). Apoptosis and pathogenesis of avian influenza A (H5N1) virus in humans. *Emerging Infectious Diseases*.

[13] Gu J., Xie Z., Gao Z. (2007). H5N1 infection of the respiratory tract and beyond: a molecular pathology study. *Lancet*.

[14] Piwpankaew Y., Monteerarat Y., Suptawiwat O., Puthavathana P., Uipresertkul M., Auewarakul P. (2010). Distribution of viral RNA, sialic acid receptor, and pathology in H5N1 avian influenza patients. *APMIS*.

[15] Sirinonthanawech N., Uiprasertkul M., Suptawiwat O., Auewarakul P. (2011). Viral load of the highly pathogenic avian influenza H5N1 virus in infected human tissues. *Journal of Medical Virology*.

[16] Sangsiriwut K., Uiprasertkul M., Payungporn S. (2018). Complete genomic sequences of highly pathogenic H5N1 avian influenza viruses obtained directly from human autopsy specimens. *Microbiology Resource Announcements*.

[17] Poddar S. K. (2002). Influenza virus types and subtypes detection by single step single tube multiplex reverse transcription-polymerase chain reaction (RT-PCR) and agarose gel electrophoresis. *Journal of Virological Methods*.

[18] World Health Organization Recommendations and laboratory procedures for detection of avian influenza A(H5N1) virus in specimens from suspected human cases. https://www.who.int/influenza/resources/documents/RecAIlabtestsAug07.pdf.

[19] Crooks G. E., Hon G., Chandonia J. M., Brenner S. E. (2004). WebLogo: a sequence logo generator. *Genome Research*.

[20] Sui J., Hwang W. C., Perez S. (2009). Structural and functional bases for broad-spectrum neutralization of avian and human influenza A viruses. *Nature Structural & Molecular Biology*.

[21] Pettersen E. F., Goddard T. D., Huang C. C. (2004). UCSF Chimera ?a visualization system for exploratory research and analysis. *Journal of Computational Chemistry*.

[22] World Health Organization Updated unified nomenclature system for the highly pathogenic H5N1 avian influenza viruses. https://cdn.who.int/media/docs/default-source/influenza/global-influenza-surveillance-and-response-system/nomenclature/updated_nomenclature_system_h5n1_avian_influenza_viruses.pdf?sfvrsn=73b9d9a6_8.

[23] Kumar S., Stecher G., Tamura K. (2016). MEGA7: molecular evolutionary genetics analysis version 7.0 for bigger datasets. *Molecular Biology and Evolution*.

[24] Mok C. K., da Guan W., Liu X. Q. (2015). Genetic characterization of highly pathogenic avian influenza A(H5N6) virus, Guangdong, China. *Emerging Infectious Diseases*.

[25] Zhang R., Chen T., Ou X. (2016). Clinical, epidemiological and virological characteristics of the first detected human case of avian influenza A(H5N6) virus. *Infection, Genetics and Evolution*.

[26] WHO CC of GISRS at CDC Atlanta (2012). H5N1 Genetic Changes Inventory: a tool for influenza surveillance and preparedness. http://www.cdc.gov/flu/avianflu/h5n1/inventory.htm.

[27] World Health Organization Human infection with avian influenza A (H5N8) – the Russian Federation. https://www.euro.who.int/en/countries/poland/news/news/2021/3/avian-influenza-ah5n8-infects-humans-in-russian-federation.

[28] To K. F., Chan P. K., Chan K. F. (2001). Pathology of fatal human infection associated with avian influenza A H5N1 virus. *Journal of Medical Virology*.

[29] de Jong M. D., Cam B. V., Qui P. T. (2005). Fatal avian influenza A (H5N1) in a child presenting with diarrhea followed by coma. *The New England Journal of Medicine*.

[30] Barbezange C., Jones L., Blanc H. (2018). Seasonal genetic drift of human influenza A virus quasispecies revealed by deep sequencing. *Frontiers in Microbiology*.

[31] Welkers M. R. A., Pawestri H. A., Fonville J. M. (2019). Genetic diversity and host adaptation of avian H5N1 influenza viruses during human infection. *Emerging Microbes & Infections*.

[32] Chaichoune K., Wiriyarat W., Thitithanyanont A. (2009). Indigenous sources of 2007-2008 H5N1 avian influenza outbreaks in Thailand. *The Journal of General Virology*.

[33] Amonsin A., Lapkuntod J., Suwannakarn K. (2010). Genetic characterization of 2008 reassortant influenza A virus (H5N1), Thailand. *Virology Journal*.

[34] Thor S. W., Nguyen H., Balish A. (2015). Detection and characterization of clade 1 reassortant H5N1 viruses isolated from human cases in Vietnam during 2013. *PLoS One*.

[35] Takayama I., Hieu T. N., Shirakura M. (2016). Novel reassortant avian influenza A(H5N1) virus in human, Southern Vietnam, 2014. *Emerging Infectious Diseases*.

[36] Suttie A., Tok S., Yann S. (2019). Diversity of A(H5N1) clade 2.3.2.1c avian influenza viruses with evidence of reassortment in Cambodia, 2014-2016. *PLoS One*.

[37] Puthavathana P., Auewarakul P., Charoenying P. C. (2005). Molecular characterization of the complete genome of human influenza H5N1 virus isolates from Thailand. *The Journal of General Virology*.

