# Supplementary material for: Full Genomic Sequences of H5N1 Highly Pathogenic Avian Influenza Virus in Human Autopsy Specimens Reveal Genetic Variability and Adaptive Changes for Growth in MDCK Cell Cultures
Source: Biomed Res Int. 2021 Jul 22;2021:3890681. doi: 10.1155/2021/3890681 (PMC8323515; doi:10.1155/2021/3890681)
Supplement: Supplementary 1 — Supplementary Figure S1: multiple alignments of the nucleotide sequences of H5N1 HPAI viral genomes in the autopsy specimens sing NGS. The genes containing variations/quasispecies are shown in open boxes. [file 3890681.f1.pdf]

PB2

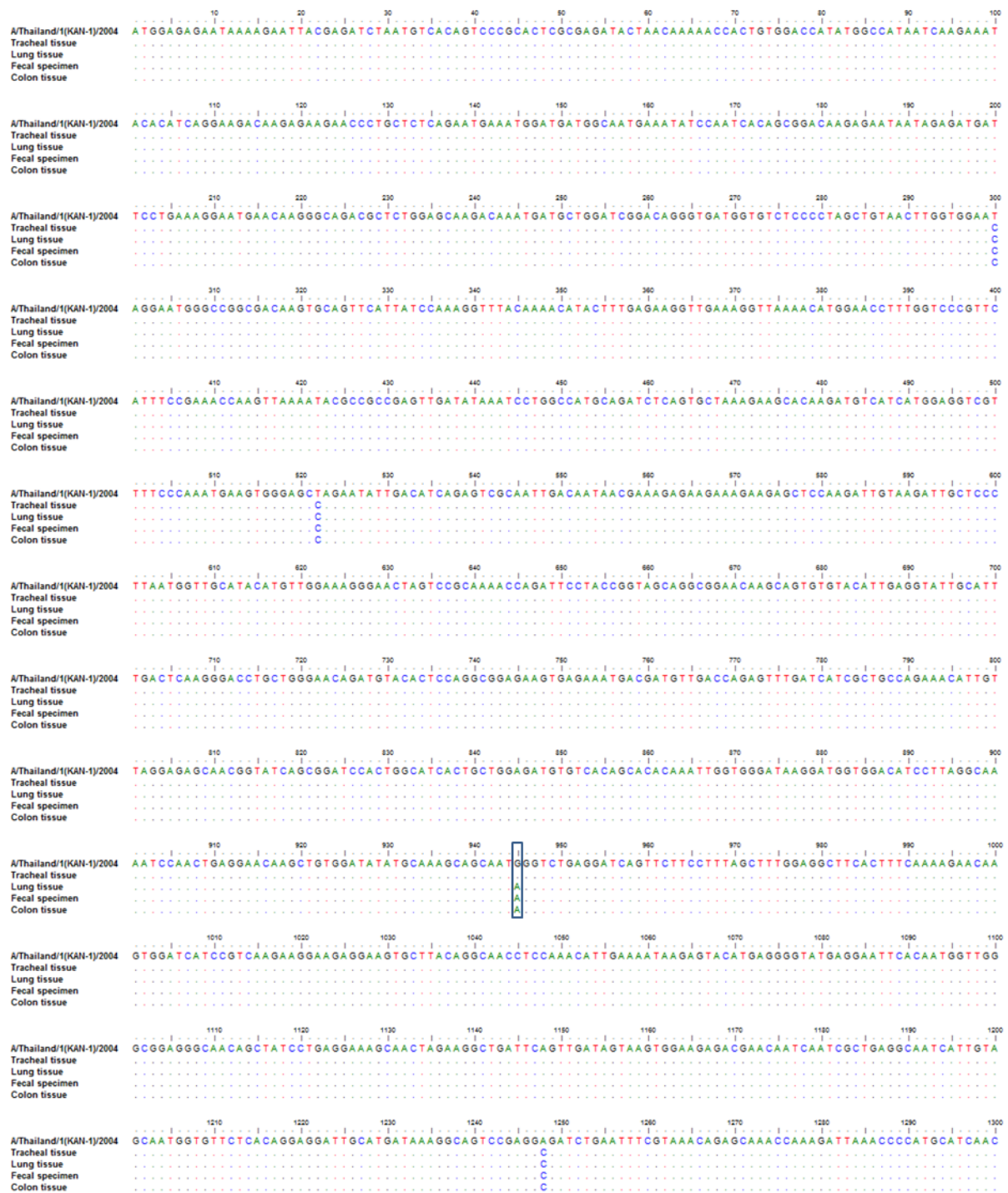

PB2 (Con't)

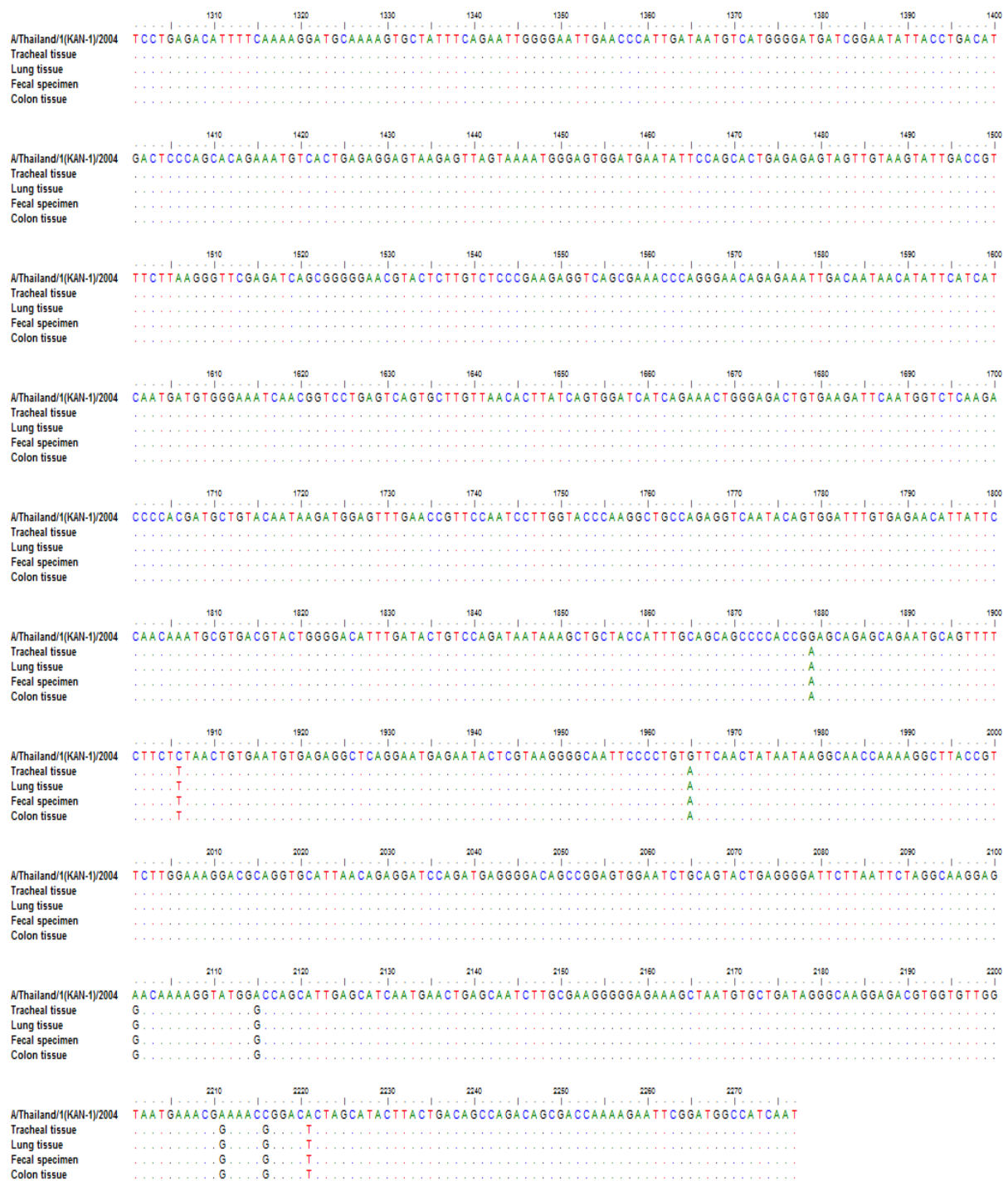

Nucleotide change at position 945 from G to A resulted in M315I amino acid change

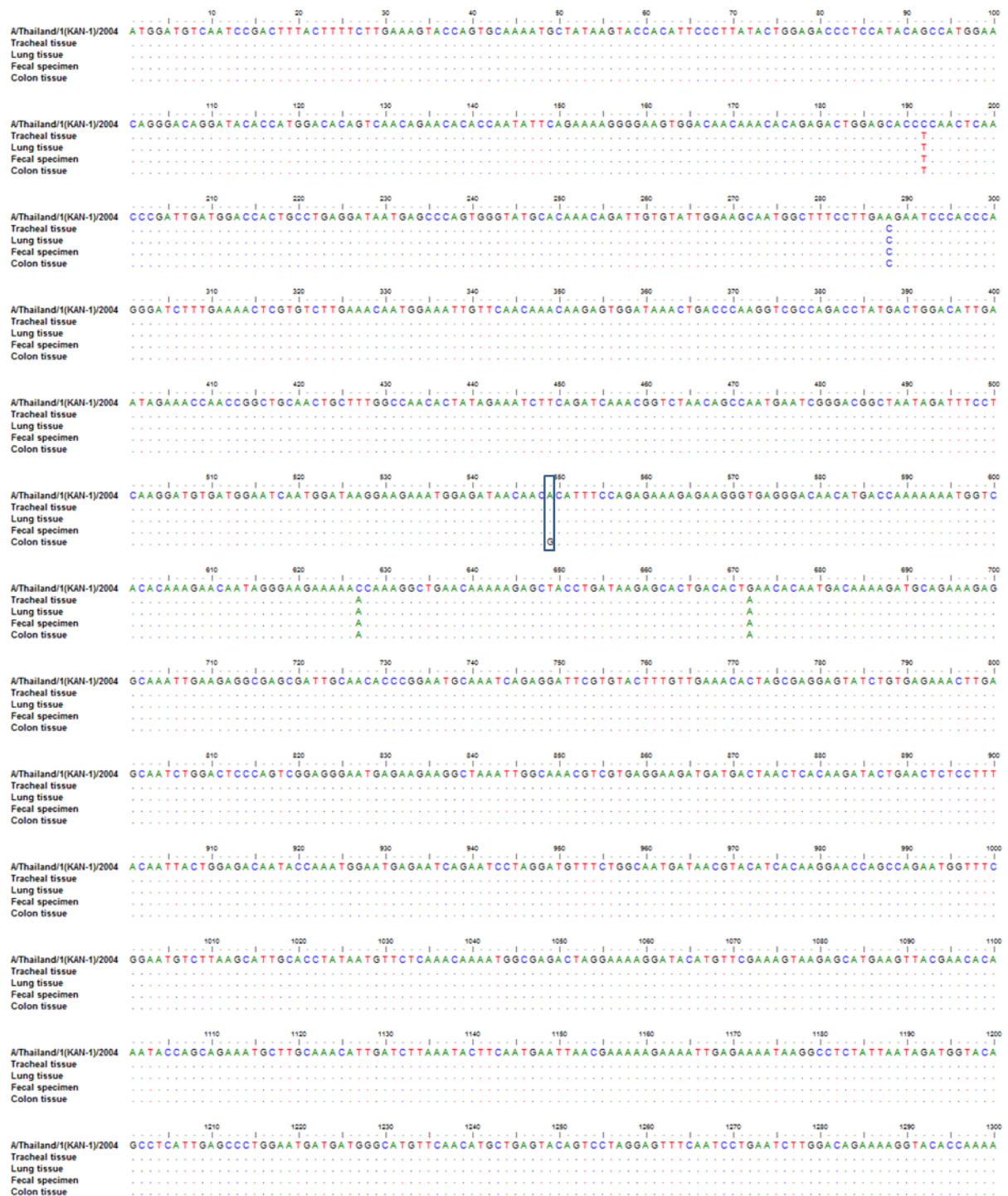

PB1 (Con't)

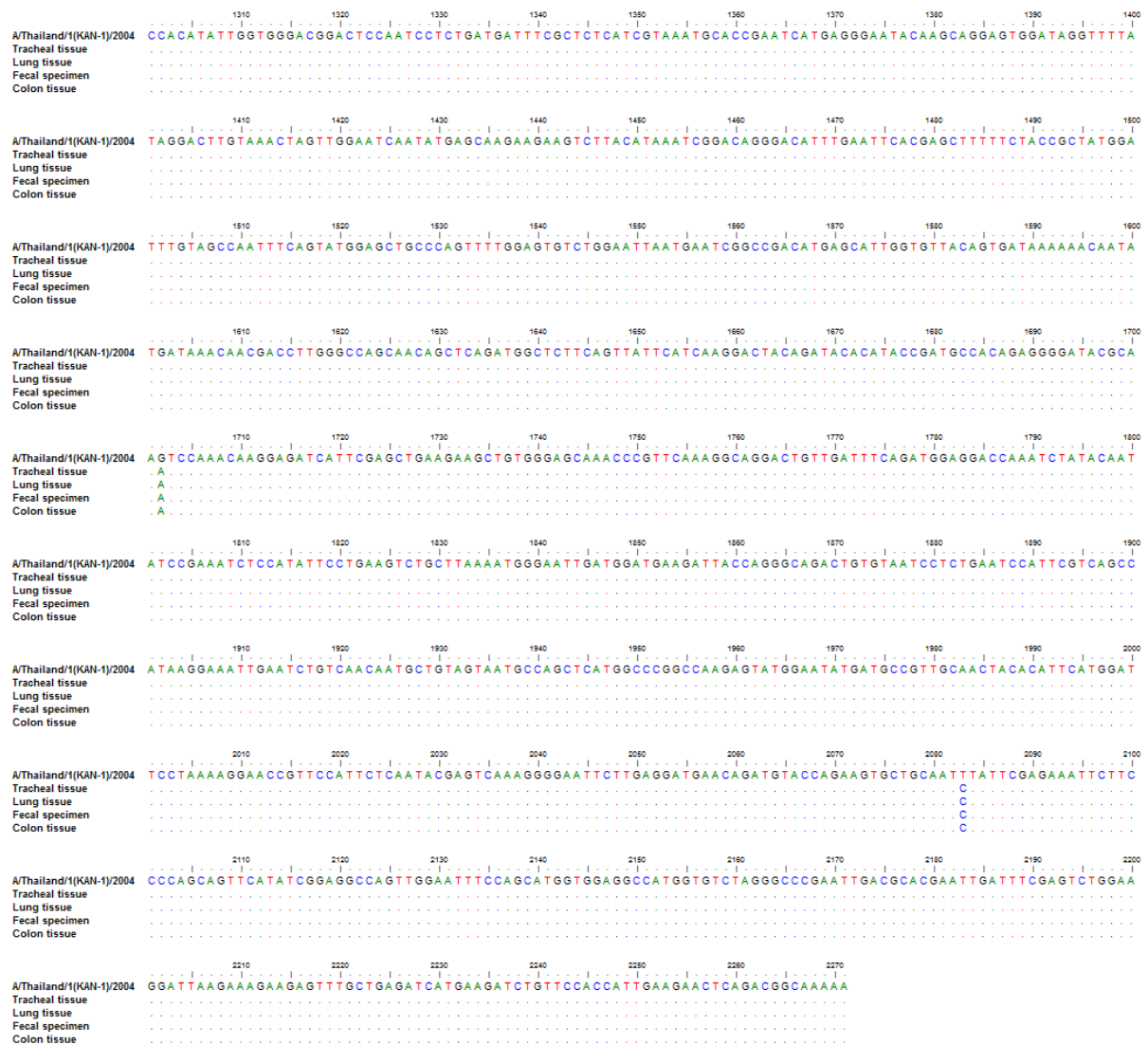

PA

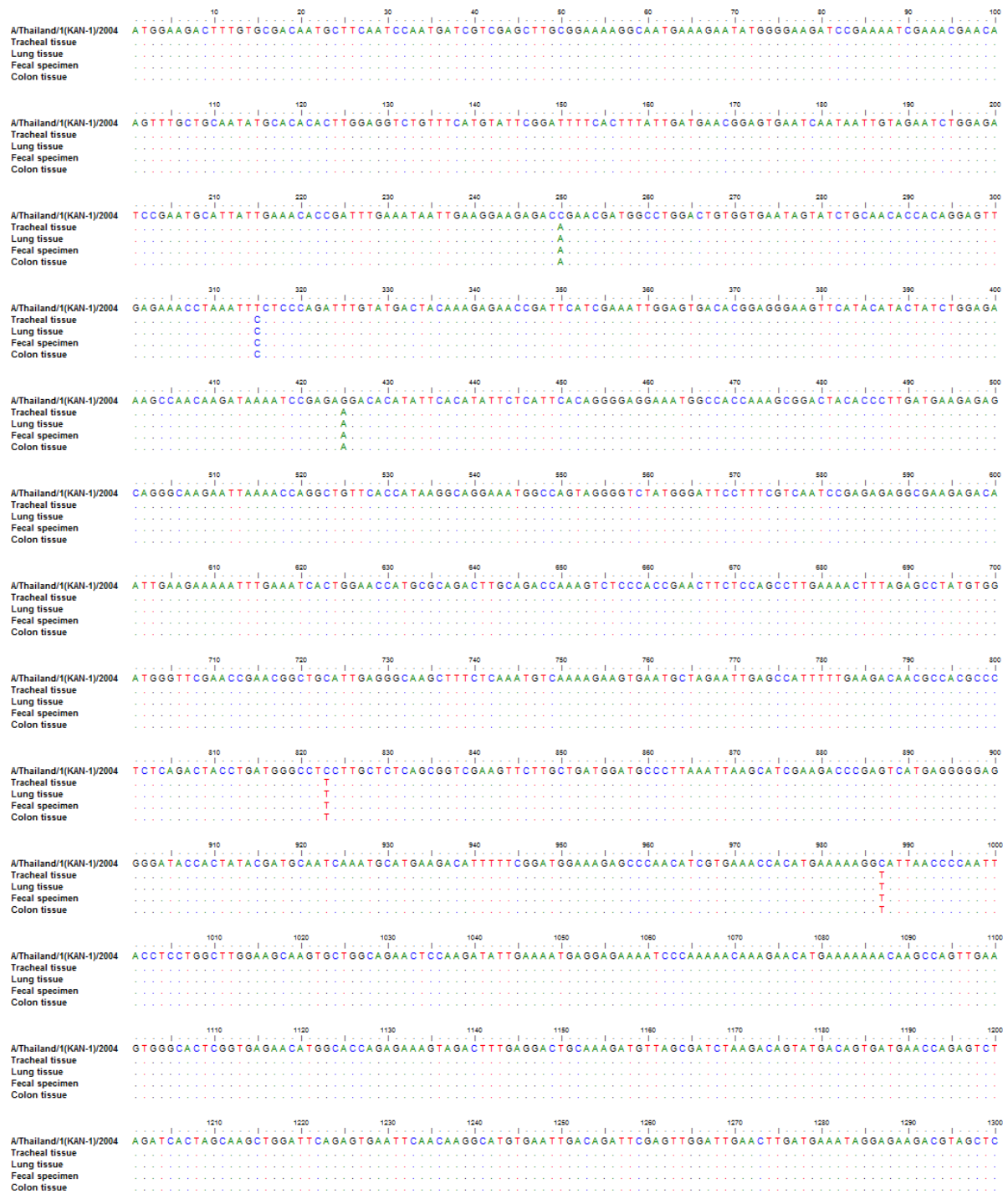

PA (Con't)

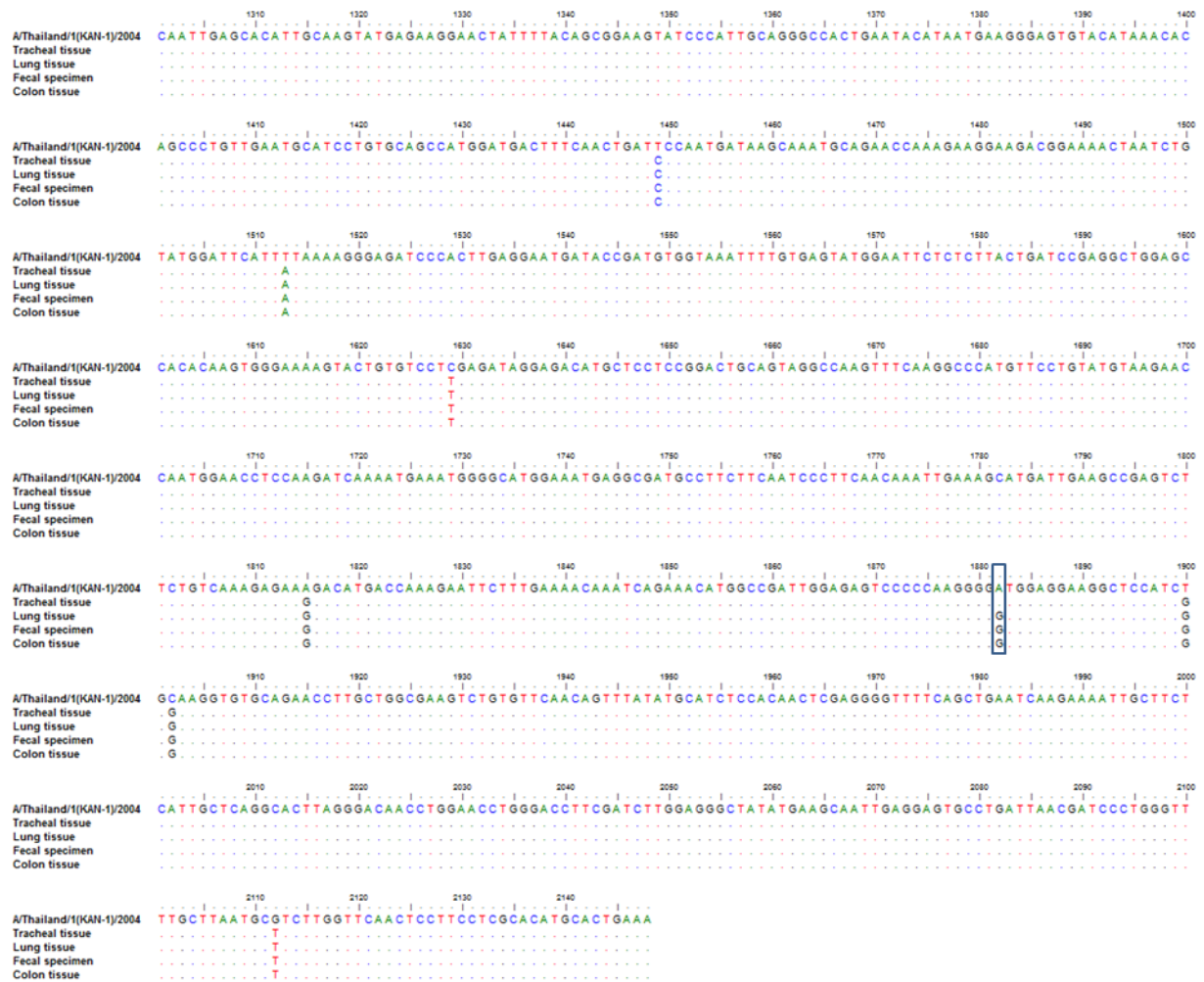

Nucleotide change at position 1882 from A to G resulted in M628V amino acid change.

HA

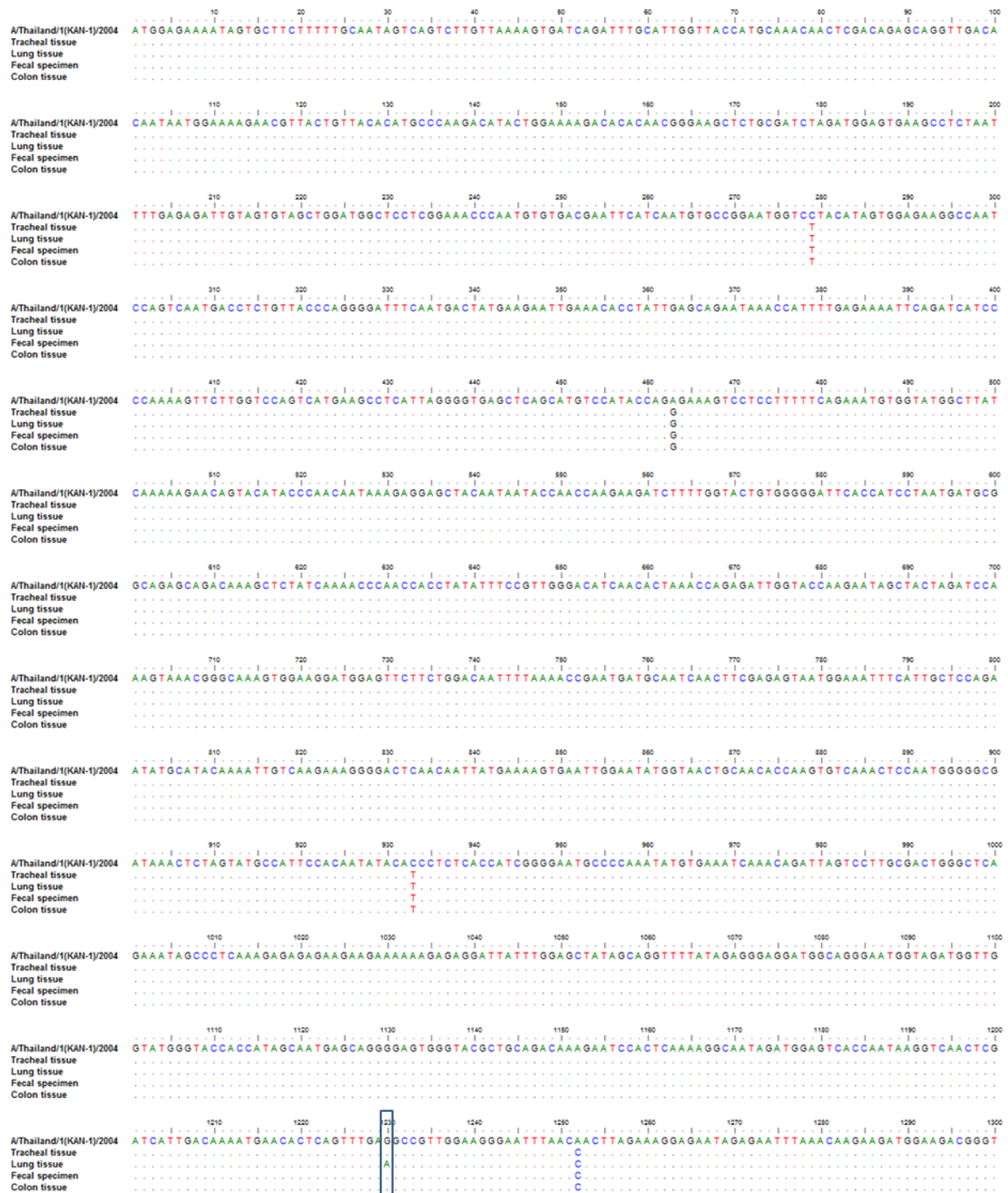

HA (Con't)

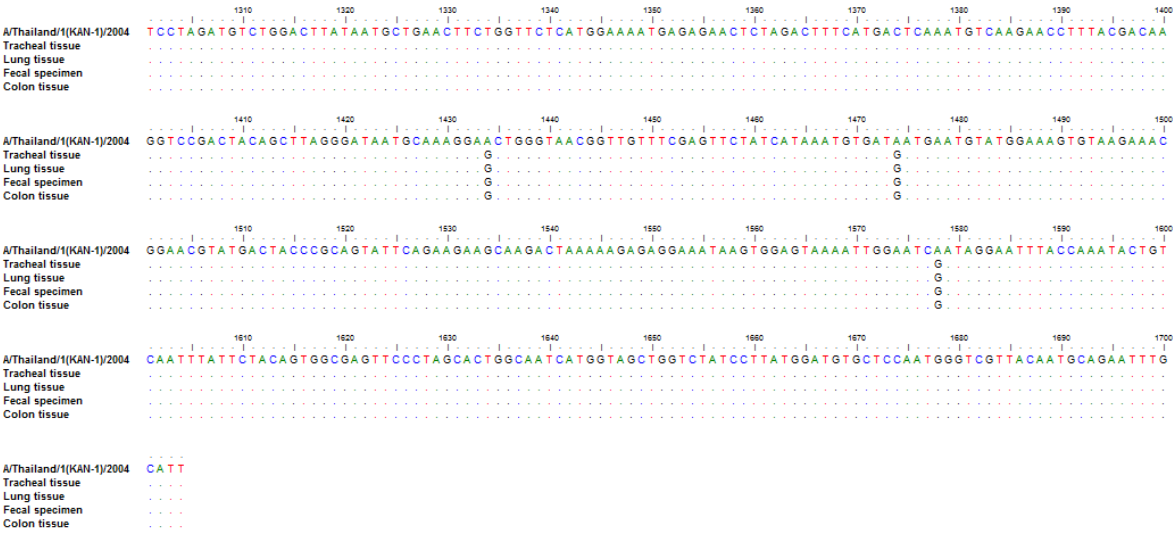

NP

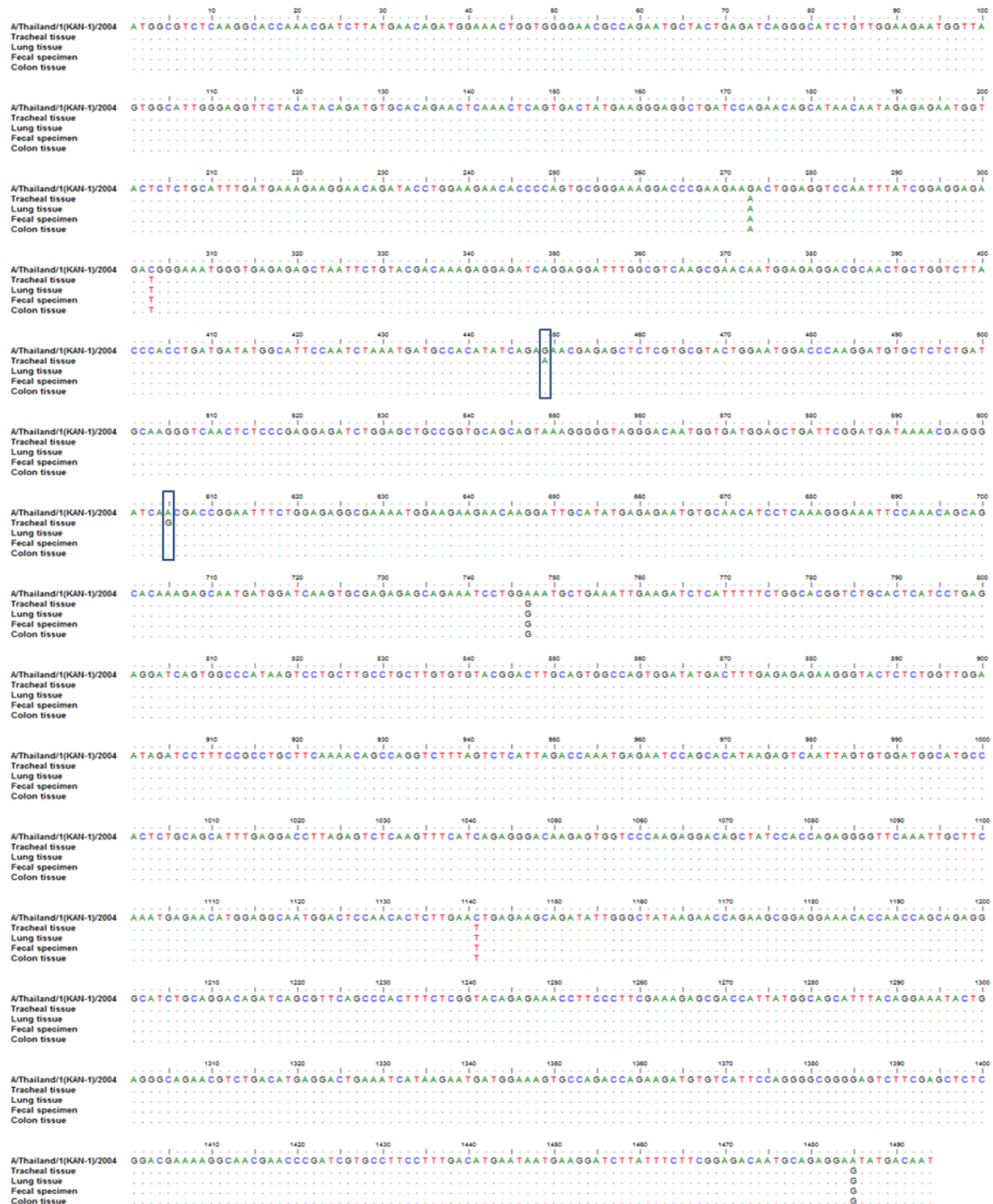

Nucleotide change at position 499 from A to G resulted in K150R amino acid change.

Nucleotide change at position 1882 from G to A resulted in S202N amino acid change.

Figure 1 displays the nucleotide sequence of the *hlyE* gene, which encodes the enterohemorrhagic *E. coli* (EHEC) enterohelminth toxin (EHEC-ETx). The sequence is presented in 100-nucleotide windows, with positions 1 to 100, 110 to 200, 210 to 300, 310 to 400, 410 to 500, 510 to 600, 610 to 700, 710 to 800, 810 to 900, 910 to 1000, 1010 to 1100, 1110 to 1200, 1210 to 1300, and 1310 to 1400. The sequence is shown for four different tissues: Tracheal tissue, Lung tissue, Fecal specimen, and Colon tissue. The sequence is color-coded by nucleotide: Adenine (A) is green, Guanine (G) is blue, Cytosine (C) is red, and Thymine (T) is black. The sequence is aligned to the reference sequence of the *hlyE* gene (GenBank accession number: F01481.1). The sequence is shown for four different tissues: Tracheal tissue, Lung tissue, Fecal specimen, and Colon tissue. The sequence is color-coded by nucleotide: Adenine (A) is green, Guanine (G) is blue, Cytosine (C) is red, and Thymine (T) is black. The sequence is aligned to the reference sequence of the *hlyE* gene (GenBank accession number: F01481.1). The sequence is shown for four different tissues: Tracheal tissue, Lung tissue, Fecal specimen, and Colon tissue. The sequence is color-coded by nucleotide: Adenine (A) is green, Guanine (G) is blue, Cytosine (C) is red, and Thymine (T) is black. The sequence is aligned to the reference sequence of the *hlyE* gene (GenBank accession number: F01481.1).

Nucleotide change at position 706 from G to A resulted in V236F amino acid change.
