# Supplementary figures and images for: Full Genomic Sequences of H5N1 Highly Pathogenic Avian Influenza Virus in Human Autopsy Specimens Reveal Genetic Variability and Adaptive Changes for Growth in MDCK Cell Cultures
Source: Biomed Res Int. 2021 Jul 22;2021:3890681. doi: 10.1155/2021/3890681 (PMC8323515; doi:10.1155/2021/3890681)

PB2

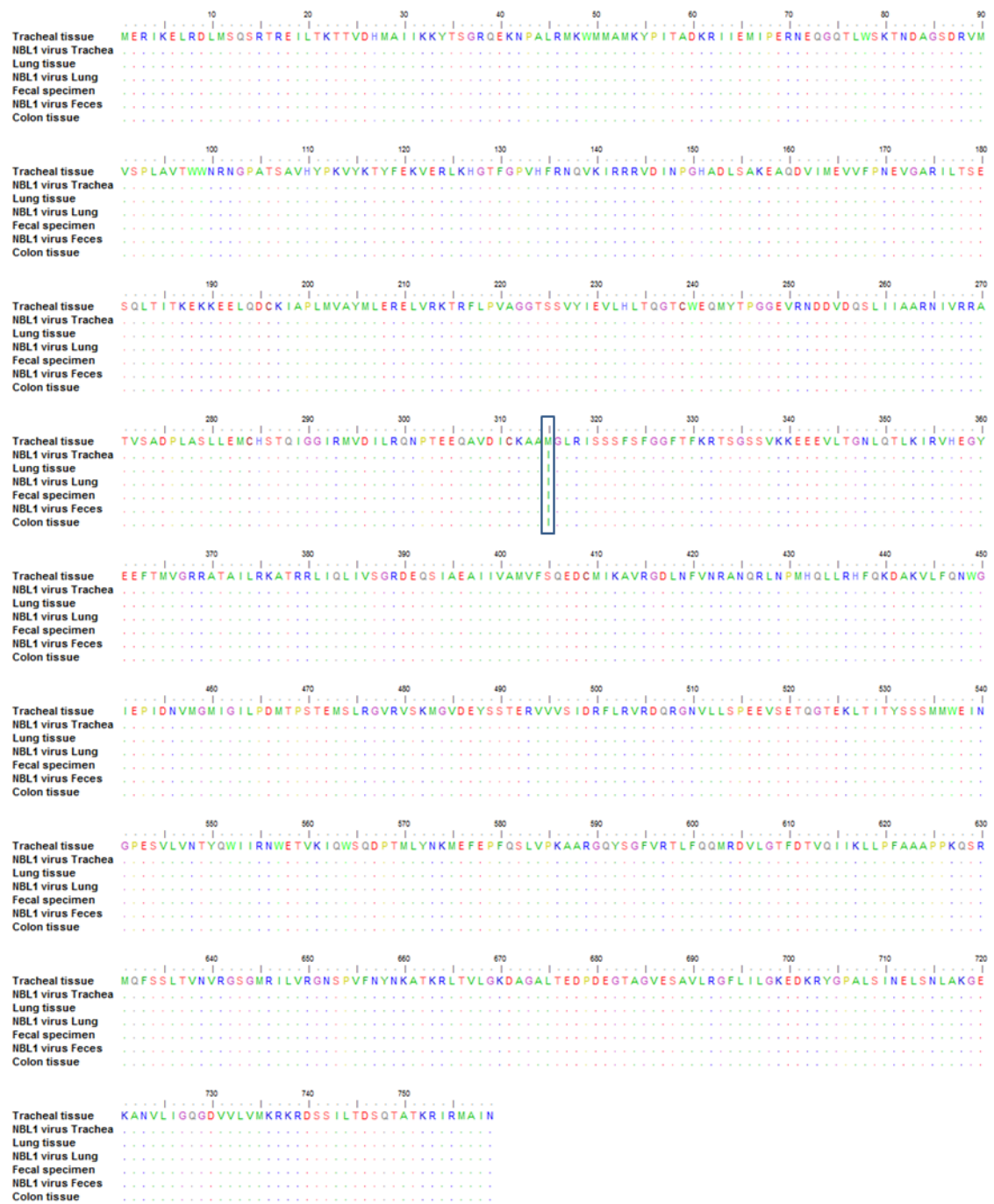

## PB1

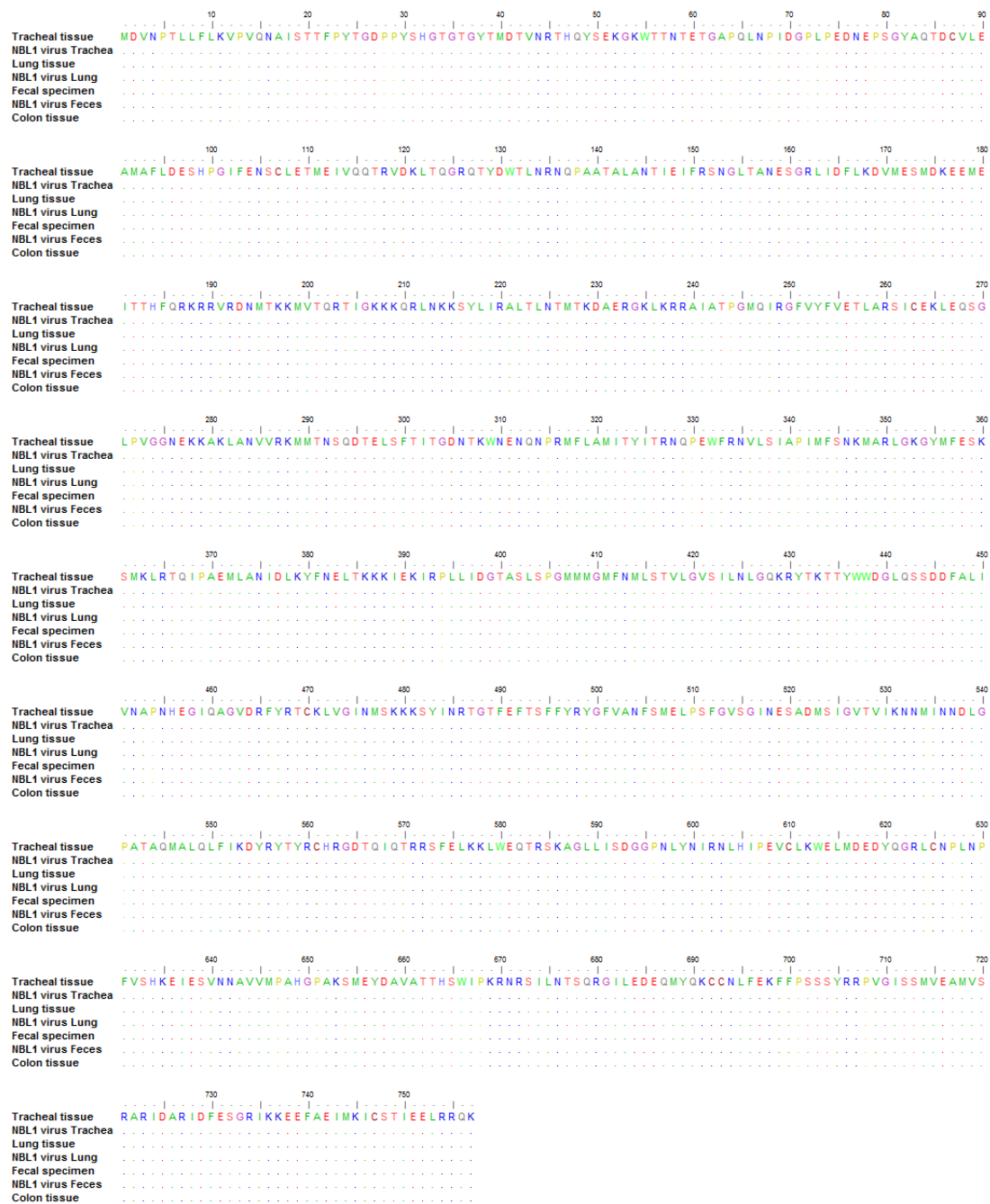

## PB1-F2

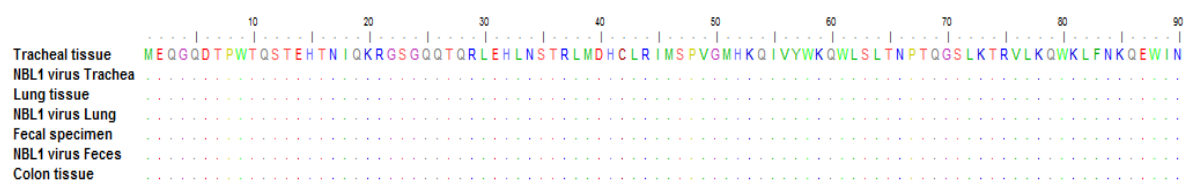

PA

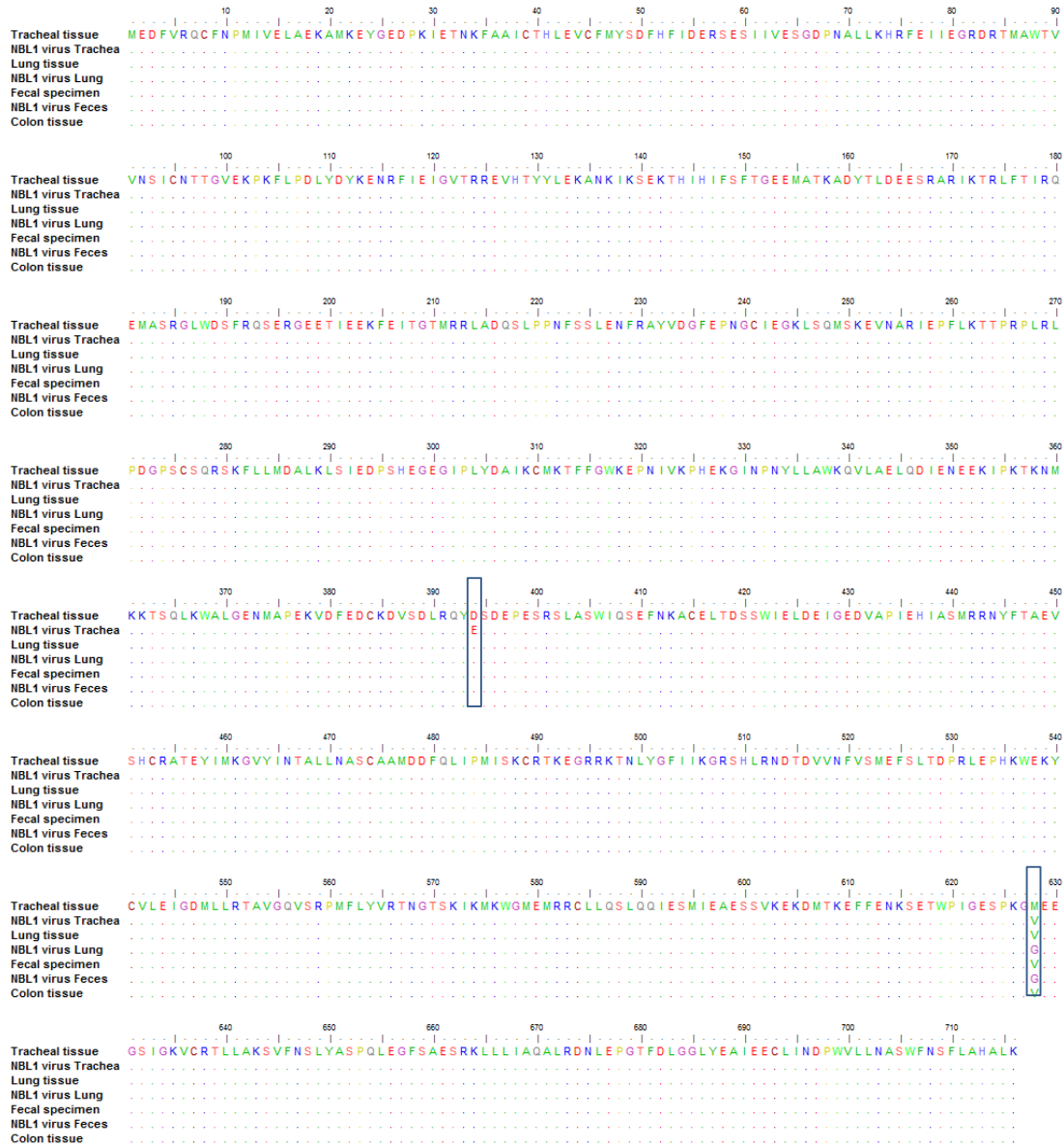

# HA

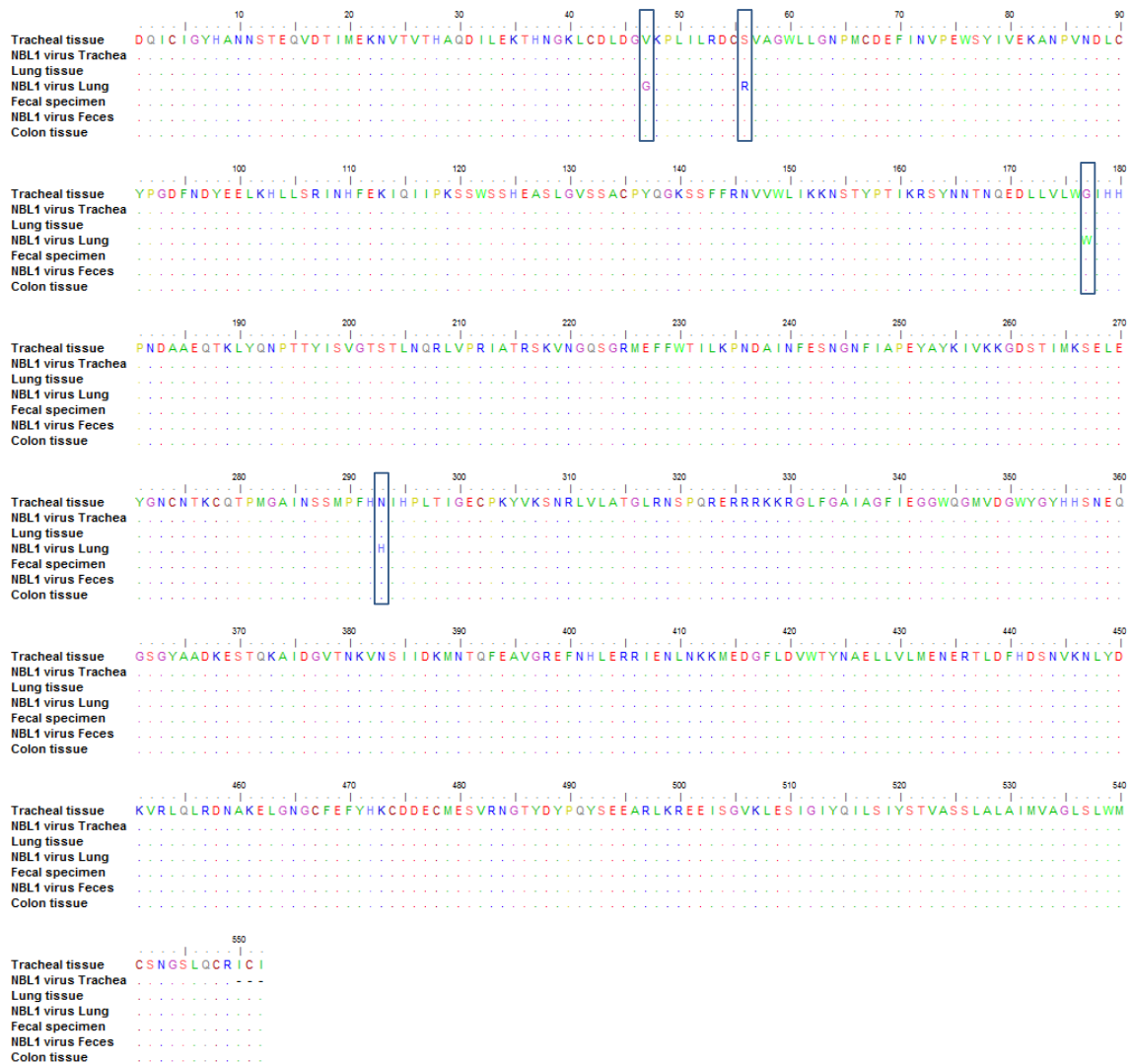

# NP

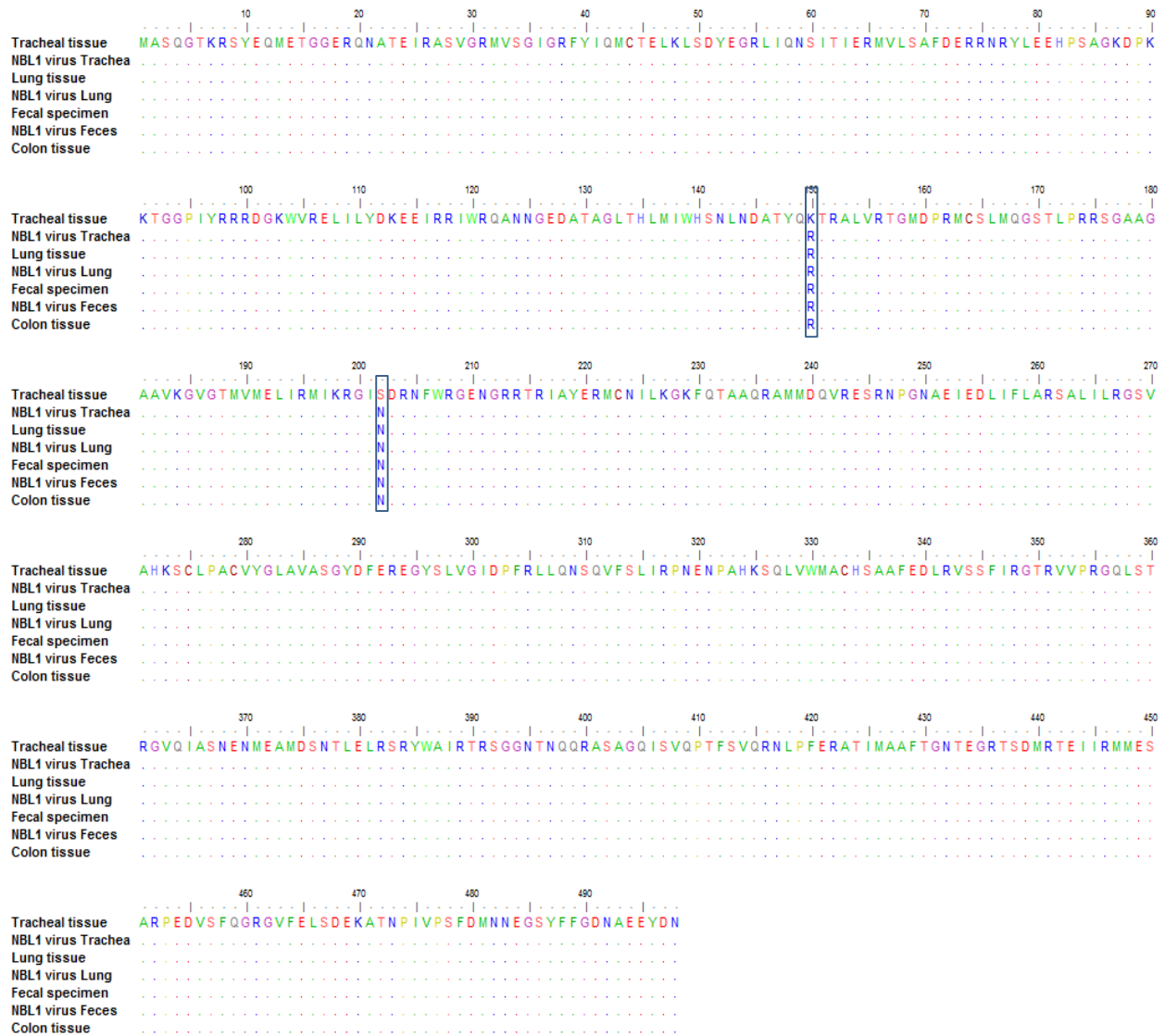

NA

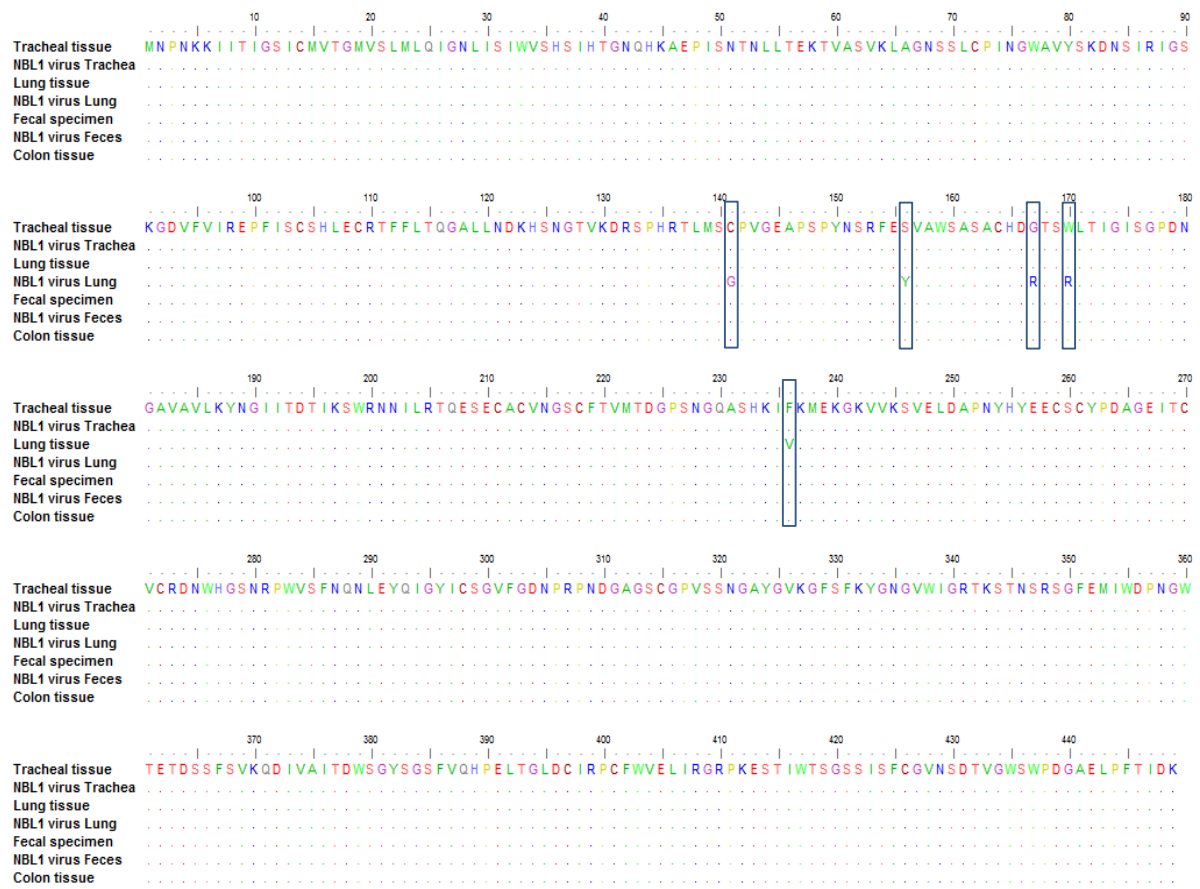

## M1

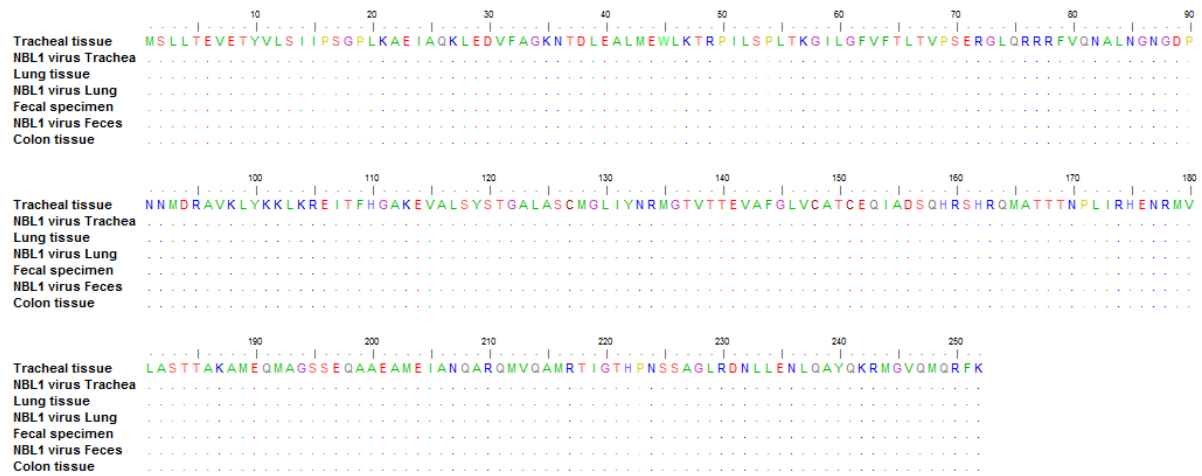

## M2

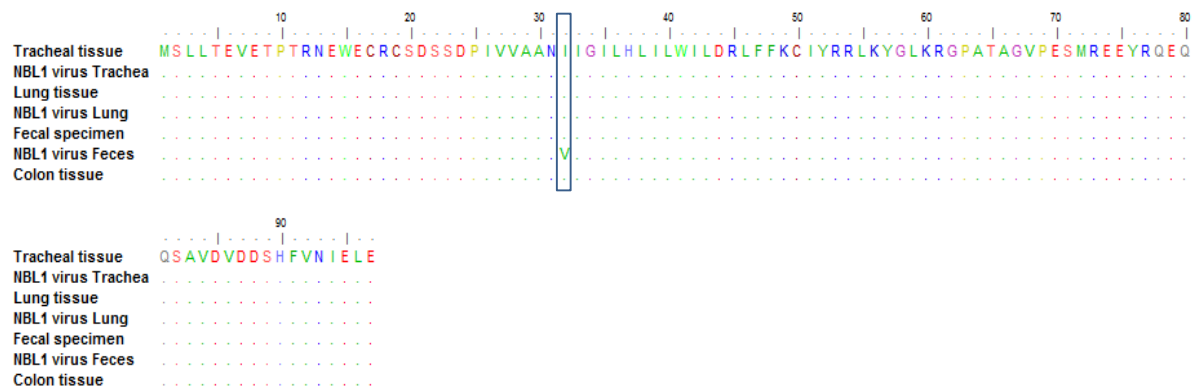

## NS1

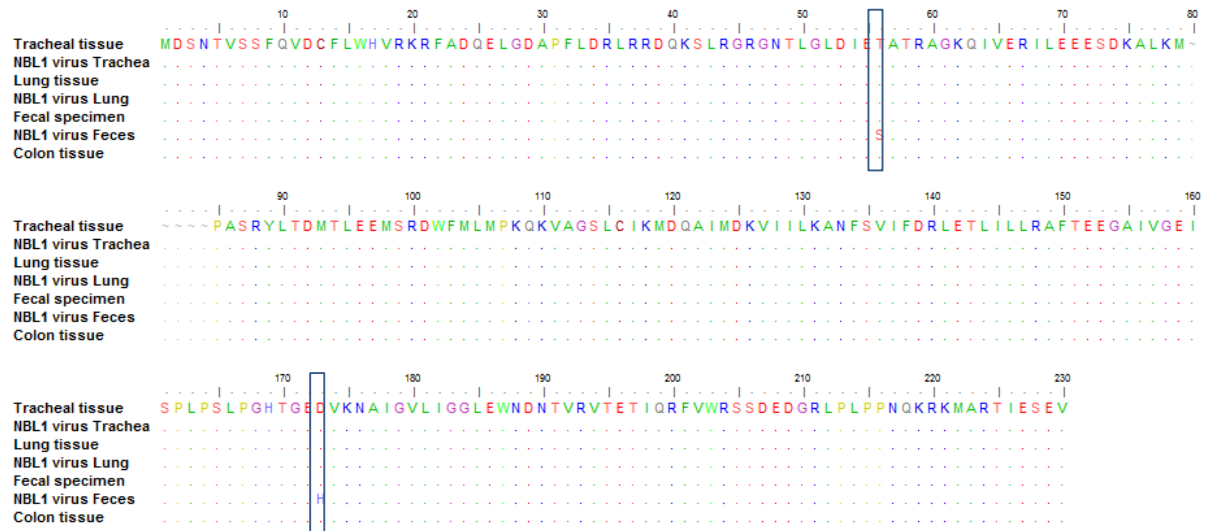

## NS2

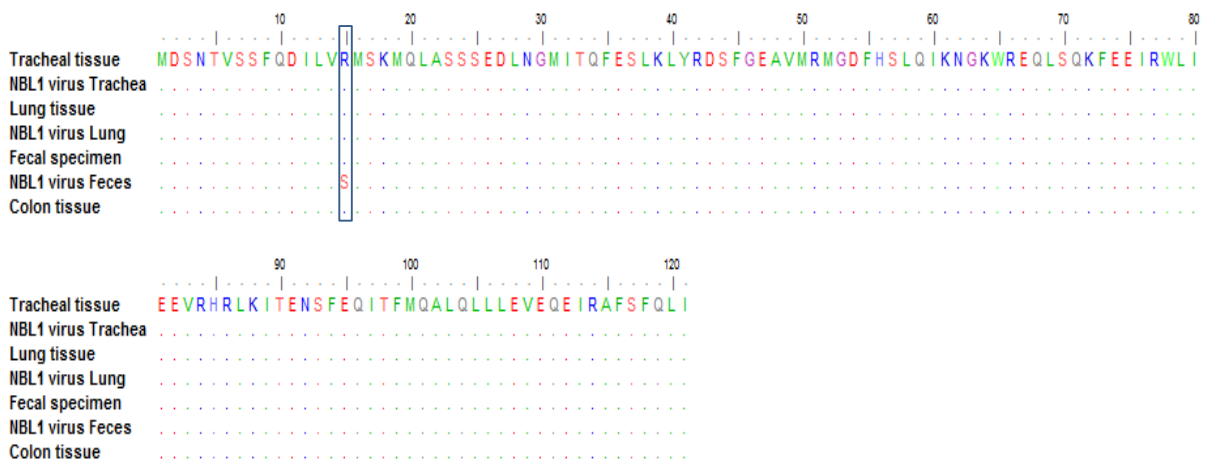

Supplement: Supplementary 2 — Supplementary Figure S2: multiple alignments of the amino acid sequences of H5N1 HPAI virus derived from the autopsy specimens and the virus isolates. The amino acid changes are shown in open boxes. [file 3890681.f2.pdf]

## PB2

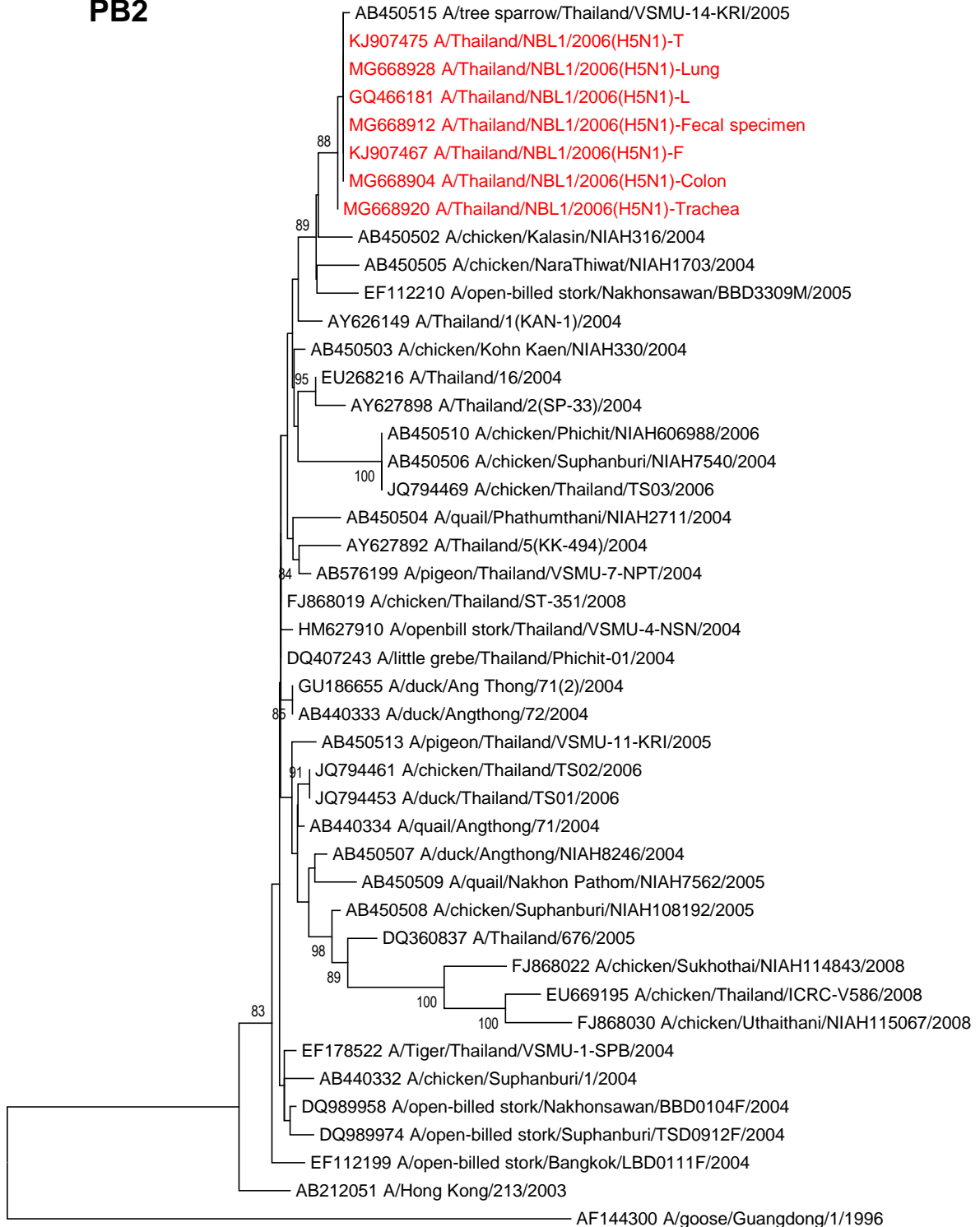

PB1

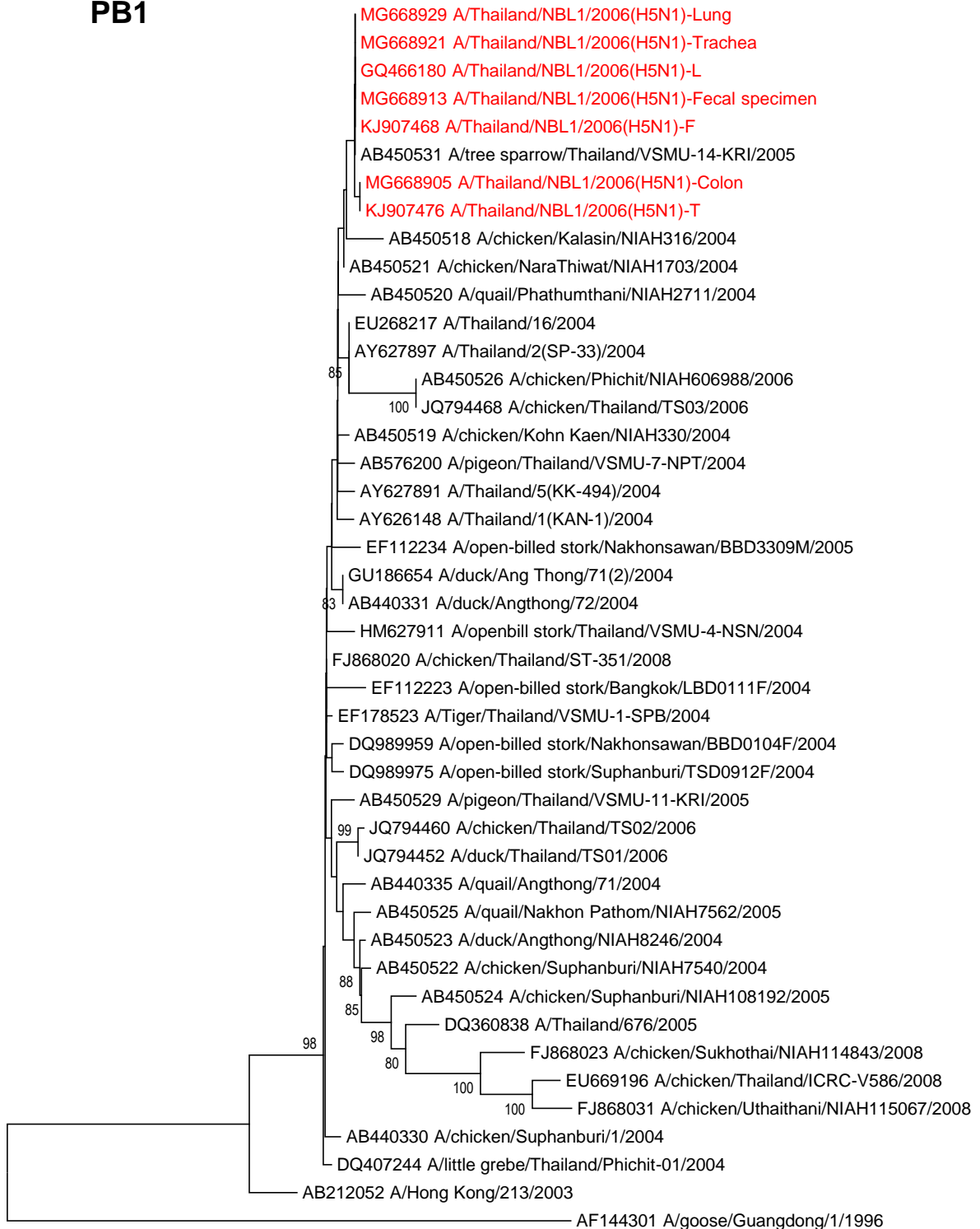

0.005

PA

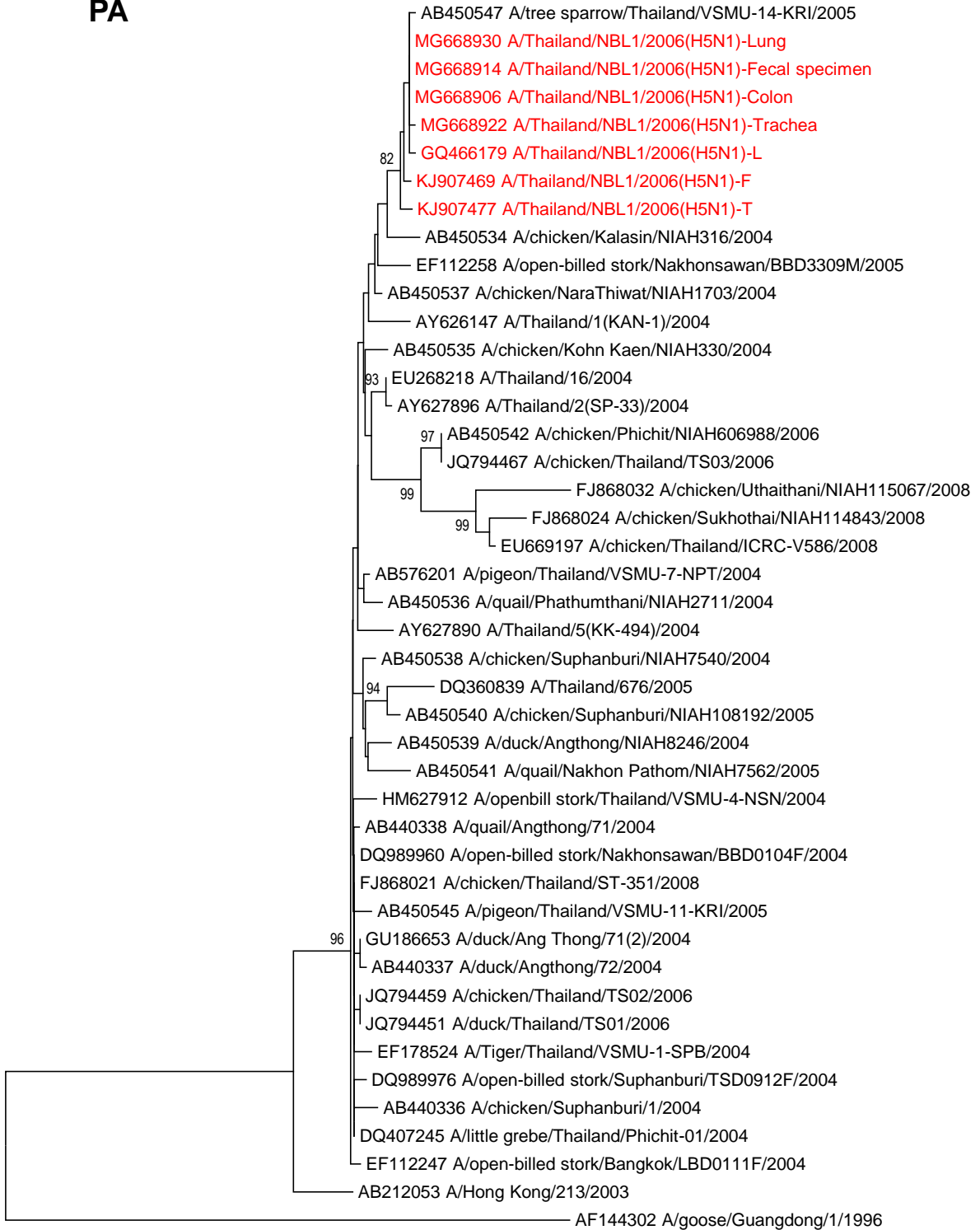

0.01

NP

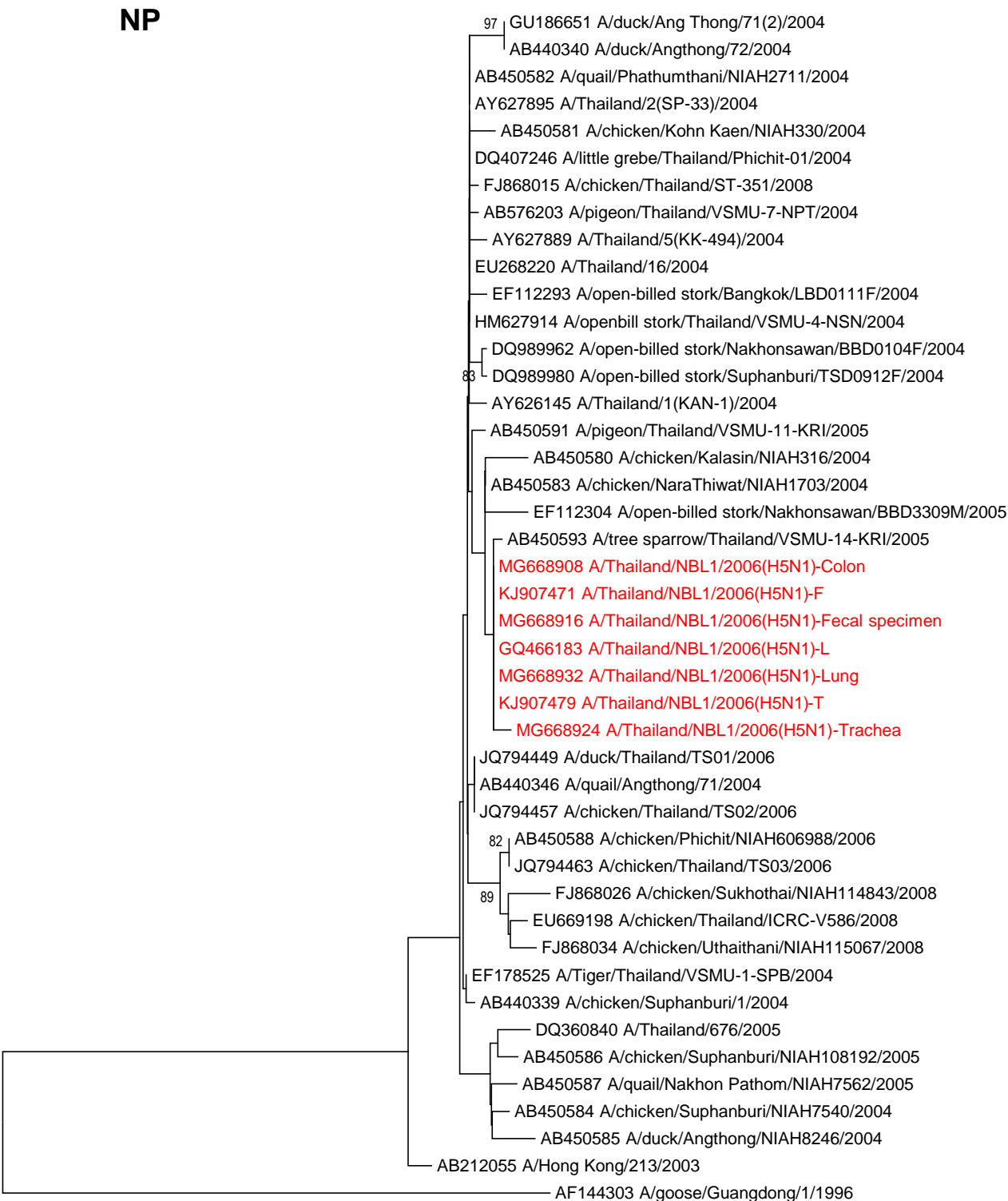

0.01

NA

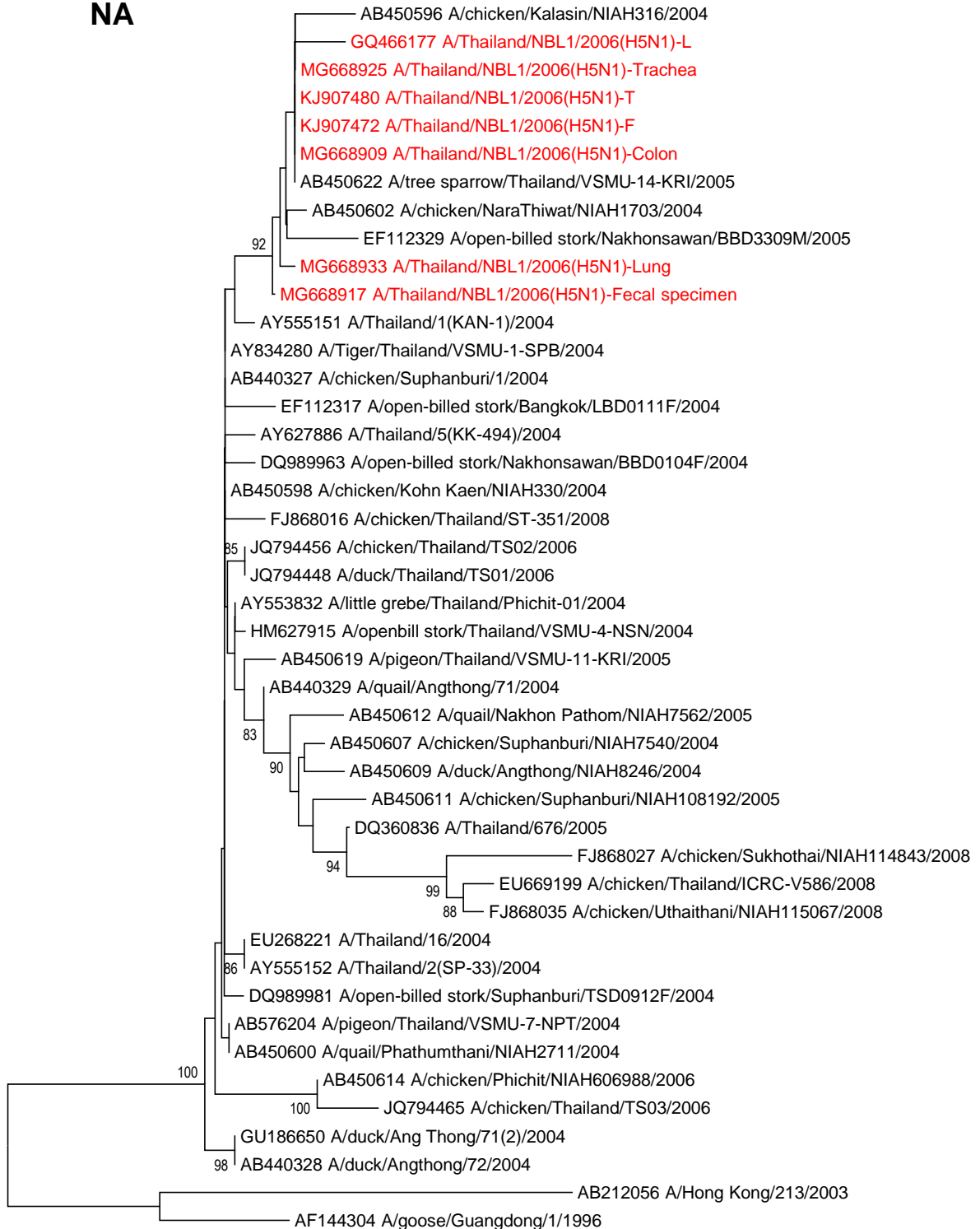

M

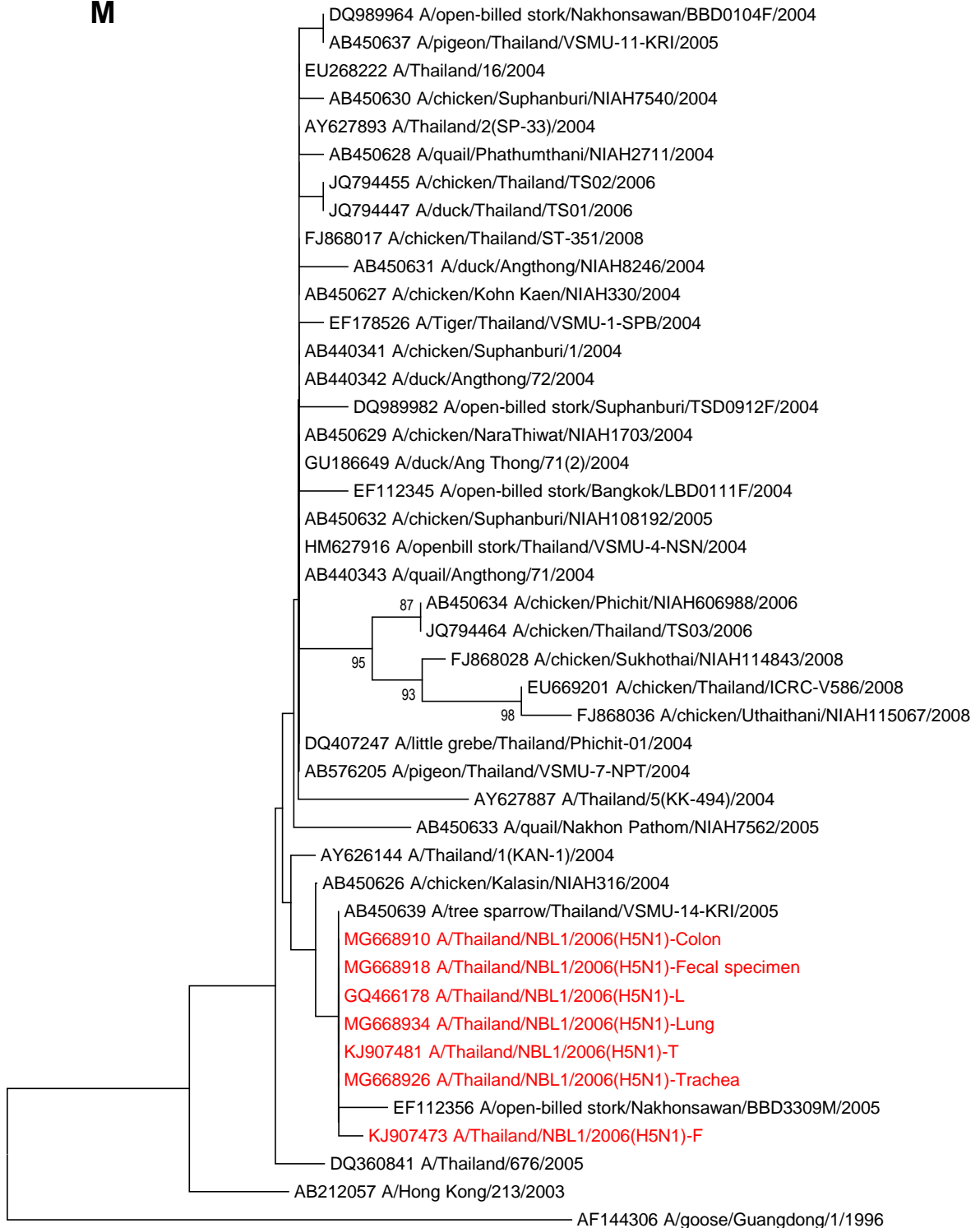

0.005

NS

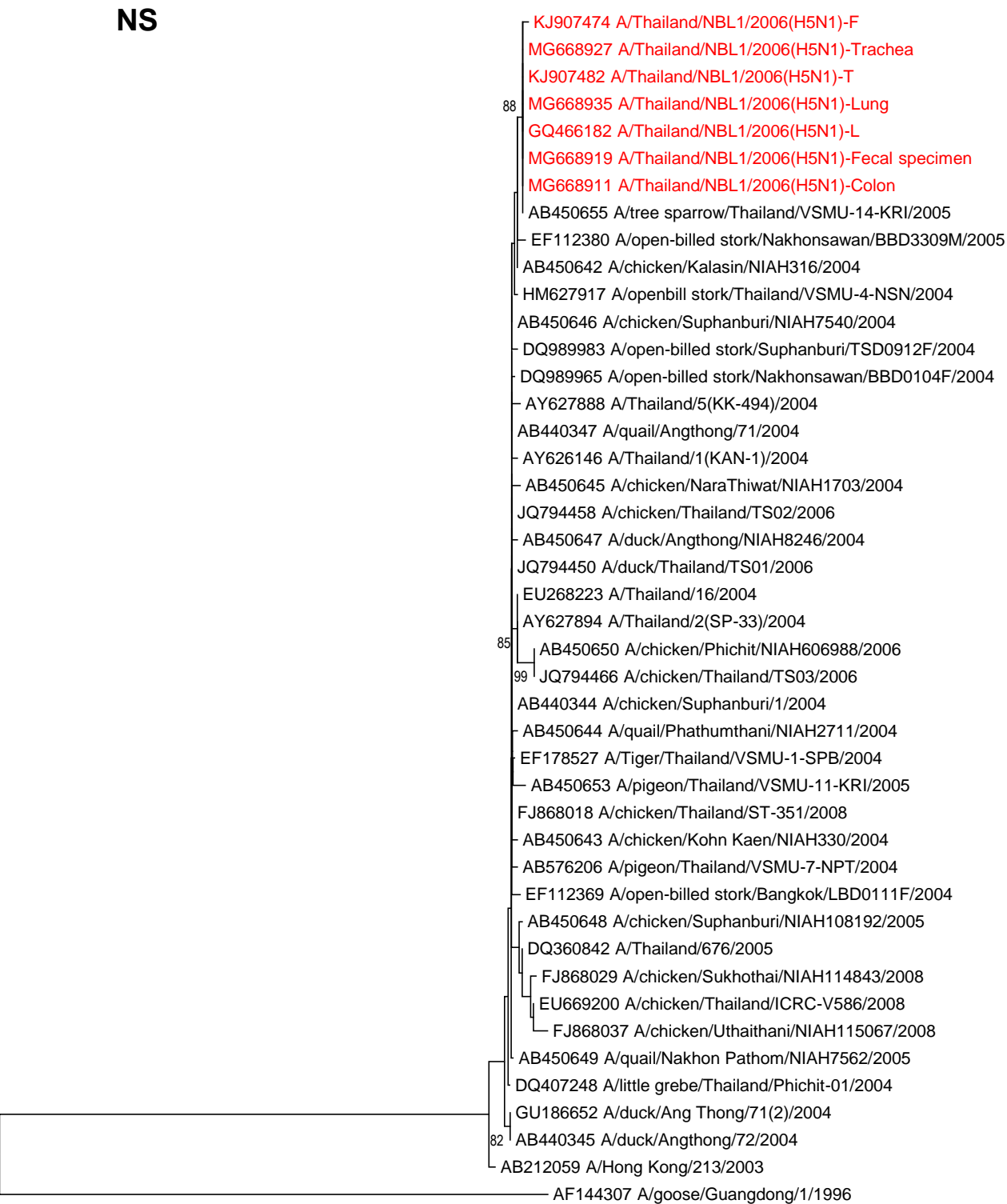

0.05

Supplement: Supplementary 3 — Supplementary Figure S3: phylogenetic analyses of an individually remaining 7 gene segments including PB2, PB1, PA, NP, NA, M, and NS from clade 1 H5N1 viruses in Thailand. [file 3890681.f3.pdf]
